# Predictor‐Assisted Nonparametric Graphical Models With Multivariate Error‐Prone Data

**DOI:** 10.1002/sim.70658

**Published:** 2026-07-07

**Authors:** Li‐Pang Chen

**Affiliations:** ^1^ Department of Statistics National Chengchi University Taipei Taiwan (ROC)

**Keywords:** bioinformatics, measurement error, network, random forest, regression calibration

## Abstract

Glioblastoma multiforme (GBM) is a highly aggressive and heterogeneous brain cancer. Emerging evidence suggests that microRNA expression profiles, together with auxiliary gene expression data, are closely associated with GBM and may provide insights into its underlying biological mechanisms. To explore these relationships, we aim to infer the network structure among microRNAs while incorporating gene expressions as auxiliary covariates. While traditional multivariate regression models are intuitive approaches, they are often inadequate in applications due to potential nonlinear relationships and measurement errors inherent in biological data. To address these challenges, we propose a novel model‐free framework for joint network inference and variable selection with multivariate responses subject to measurement error. Our method integrates random forests to model marginal response‐covariate relationships with built‐in error correction, and extends the graphical lasso to recover the conditional dependency structure among microRNAs. This approach is easy for the implementation and offers robustness and flexibility in complex, error‐prone biological datasets. Simulation studies and GBM data analysis demonstrate that the proposed method outperforms existing techniques in accurately identifying network structures and selecting informative covariates, offering new insights into the molecular architecture of GBM.

## 1 Introduction

Modeling the relationship between multiple response variables and high‐dimensional covariates is a central problem in multivariate statistics and machine learning. Such modeling enables researchers to understand and predict complex biological systems. A motivating application arises in the study of glioblastoma multiforme (GBM), an aggressive and heterogeneous form of brain cancer. Data from The Cancer Genome Atlas (TCGA) provide rich molecular profiles, including 534 microRNA expression levels and 11,861 gene expression measurements, both treated as continuous variables [1, 2]. In this study, we aim to investigate the relationship between microRNA expression levels, denoted as the response vector $Y\in {\mathbb{R}}^{m}$, and gene expression profiles, treated as covariates $X\in {\mathbb{R}}^{p}$, to better understand the regulatory mechanisms underlying GBM, where the dimensions m and p are fixed positive integers.

A widely used approach for modeling the relationship between multivariate responses Y and covariates X is the multivariate linear regression model [3, 4]. While intuitive and computationally convenient, this model faces several challenges when applied to high‐dimensional bioinformatics data. One major issue is the presence of non‐informative gene expressions, which, if not properly excluded, can degrade estimation accuracy and increase computational burden. To address this, various variable selection methods have been proposed (e.g., [5, 6]). However, many of these approaches assume linear relationships and may struggle in complex, nonlinear biological systems.

In addition to variable selection, another important characteristic of bioinformatics data is the potential network structure among response variables, such as microRNAs. These structures are often represented by a graph 𝒢=(𝒱,ℰ), where 𝒱 is the set of nodes corresponding to microRNAs and ℰ is the set of edges indicating conditional dependencies among nodes. Understanding such networks can yield biologically meaningful insights. Classical methods like the graphical lasso (glasso, [7]), $\ell_{1}$‐penalized precision matrix estimation (CLIME, [8]), and others in [9] are effective in estimating sparse precision matrices. However, most of these methods are unsupervised and do not incorporate covariate information. Supervised extensions, such as those proposed by [10, 11], attempt to integrate covariates through penalized likelihood formulations. However, these extensions continue to rely on linear modeling assumptions, which may limit their applicability in capturing the complexity of real biological systems.

Another major challenge in high‐throughput biological data is measurement error, which may result from experimental noise, batch effects, or sample heterogeneity (e.g., [12, 13]). In practice, we do not observe the true variables Y and X directly; instead, we observe surrogate versions W and Z, respectively. Although actual measurement errors in biological contexts can be inherently complex, nonlinear, or multiplicative, the classical additive measurement error model (e.g., [14]) is widely adopted in practice, as it can serve as a reasonable first‐order approximation to more sophisticated nonlinear structures. Specifically, additive measurement error models are formulated as

(1) $$\begin{array}{l} \text{W}=\text{Y}+\delta \;\;\text{and}\;\;\text{Z}=\text{X}+\eta , \end{array}$$

where δ and η are error terms with mean zero and covariance matrices $\sum_{\delta }$ and $\sum_{\eta }$, respectively. Moreover, we assume that the errors δ and η are independent of each other. Moreover, δ is independent of Y, and η is independent of X. Ignoring measurement errors can lead to unreliable results, such as inaccurate network detection or variable selection.

While several methods account for measurement error in unsupervised settings (e.g., [13, 15]), they do not incorporate covariate information. Conversely, recent work by [16] addresses covariate measurement error in multivariate regression with variable selection, but their method neither infers network structures among responses nor accommodates nonlinear effects. These limitations point to a methodological gap in jointly addressing variable selection, network estimation, and measurement error within a flexible framework.

To overcome these challenges, we propose a model‐free framework that integrates variable selection, network estimation, and measurement error correction for multivariate response analysis. Our method adopts a two‐stage strategy. In the first stage, we apply random forests to individually regress each microRNA on gene expression covariates, while correcting for measurement error. Variable importance scores are used to select informative gene expressions. In the second stage, we compute residuals from the fitted models and assess the dependence structure among microRNAs using distance correlation (DC), followed by applying the graphical lasso to estimate the graphical structure. This model‐free approach accommodates nonlinear relationships, corrects for measurement error, and produces interpretable and biologically meaningful network structures, as demonstrated in both simulation studies and real GBM data analysis.

The remainder is organized as follows. In Section 2, we introduce the estimation method to perform variable selection and network detection for nonlinear models. In Section 3, we conduct simulation studies to assess the performance of the proposed methods. In Section 4, we apply the proposed method to analyze a real dataset. A general discussion is presented in Section 5. Numerical implementations are written by R programming language, and the corresponding code is placed at the GitHub https://github.com/lchen723/RF_Graph

## 2 Methodology

In this section, we introduce the proposed estimation procedure in the following subsections. Let n denote the sample size. For i=1,⋯,n, let ${\text{W}}_{i}$ and ${\text{Z}}_{i}$ denote the independent and identically distributed (i.i.d.) samples of W and Z, respectively.

### 2.1 Measurement Error Correction

In this step, we introduce the regression calibration method to deal with measurement error in Y and X, since we do not impose any model assumption between Y and X. The key idea of the regression calibration method is to adopt the conditional expectation to derive the “working variables,” which can be used to replace error‐prone variables directly and make the approximation to the unobserved responses and covariates, yielding a flexible way to make the correction.

Specifically, we consider ${\text{W}}^{*}≜E(\text{Y}|\text{W})$ and ${\text{Z}}^{*}≜E(\text{X}|\text{Z})$. By the measurement error structure (1), it follows that $\mu_{W}≜E({\text{W}}^{*})=E(\text{Y})$ and $\mu_{Z}≜E({\text{Z}}^{*})=E(\text{X})$. Moreover, the former identity implies $E\{E(\text{Y}|{\text{Z}}^{*})\}=E\{E({\text{W}}^{*}|{\text{Z}}^{*})\}$, which yields $E\{E(\text{Y}|{\text{Z}}^{*})-E({\text{W}}^{*}|{\text{Z}}^{*})\}=\text{0}$ with 0 being the zero vector. On the other hand, by the nondifferential measurement error mechanism (e.g., [14, Section 2.5]), we note that $E(\text{Y}|{\text{Z}}^{*})=E\{E(\text{Y}|{\text{Z}}^{*},\text{X})|{\text{Z}}^{*}\}=E\{E(\text{Y}|\text{X})|{\text{Z}}^{*}\}$. Utilizing the property that $E({\text{W}}^{*}|{\text{Z}}^{*})=E\{E({\text{W}}^{*}|{\text{Z}}^{*})|{\text{Z}}^{*}\}$, we establish that

$$\begin{array}{ll} \text{0} & =E\{E(\text{Y}|{\text{Z}}^{*})-E({\text{W}}^{*}|{\text{Z}}^{*})\}\\ & =E\left[E\{E(\text{Y}|\text{X})-E({\text{W}}^{*}|{\text{Z}}^{*})|{\text{Z}}^{*}\}\right]\\ & =E\{E(\text{Y}|\text{X})-E({\text{W}}^{*}|{\text{Z}}^{*})\}. \end{array}$$

This result implies that the discrepancy between the latent conditional mean response E(Y|X) and its regression‐calibrated counterpart $E({\text{W}}^{*}|{\text{Z}}^{*})$ has zero expectation. Hence, the regression calibration procedure preserves the latent mean structure at the population level, supporting the use of ${\text{W}}^{*}$ and ${\text{Z}}^{*}$ as corrected surrogate variables.

To express the conditional expectation E(Y|W) and E(X|Z), we adopt the best linear approximation (e.g., [14]), which are respectively given by

(2) $$E(Y|W)=\mu_{W}+{\left(\sum_{W}-\sum_{\delta }\right)}^{⊤}\sum_{W}^{-1}(W-\mu_{W})$$

and

(3) $$E(X|Z)=\mu_{Z}+{\left(\sum_{Z}-\sum_{\eta }\right)}^{⊤}\sum_{Z}^{-1}(Z-\mu_{Z}),$$

where $\sum_{W}$ and $\sum_{Z}$ are covariance matrices of W and Z, respectively. We treat $\sum_{\delta }$ and $\sum_{\eta }$ as known matrices temporarily, and we estimate $\mu_{W}$, $\mu_{Z}$, $\sum_{W}$ and $\sum_{Z}$ empirically by the observed data ${\text{W}}_{i}$ and ${\text{Z}}_{i}$, and denote the corresponding estimators as ${\hat{\mu }}_{W}$, ${\hat{\mu }}_{Z}$, ${\hat{\sum }}_{W}$ and ${\hat{\sum }}_{Z}$. Then, for each subject i=1,…,n, (2) and (3) yield the following estimated calibrated scores for the responses and the covariates, respectively:

$$\begin{array}{l} {\hat{W}}_{i}≜{\hat{\mu }}_{W}+({\hat{\sum }}_{W}-\sum_{\delta }){\hat{\sum }}_{W}^{-1}(W_{i}-{\hat{\mu }}_{W}) \end{array}$$

and

$$\begin{array}{l} {\hat{Z}}_{i}≜{\hat{\mu }}_{Z}+({\hat{\sum }}_{Z}-\sum_{\eta }){\hat{\sum }}_{Z}^{-1}(Z_{i}-{\hat{\mu }}_{Z}). \end{array}$$

Thus, the resulting corrected dataset is given by $D≜\{\{{\hat{W}}_{i},{\hat{Z}}_{i}\}:i=1,\ldots ,n\}$.

In applications, the measurement error covariance matrices $\sum_{\delta }$ and $\sum_{\eta }$ are usually unknown. If auxiliary information is unavailable, then one should specify several values by the research experience, background knowledge of the dataset, or sensitivity analyses. If one wishes to estimate $\sum_{\delta }$ and $\sum_{\eta }$, then auxiliary datasets are required. Since the procedures are similar, we focus the discussion on the estimation of $\sum_{\eta }$; the same strategies hold for $\sum_{\delta }$.

In the presence of repeated measurements for covariates, suppose that ${\text{Z}}_{ir}$ is available for i=1,…,n and $r=1,\ldots ,n_{i}$. Then, we characterize ${\text{Z}}_{ir}$ and ${\text{X}}_{i}$ by the measurement error model (1). Following the discussion in [14], $\sum_{\eta }$ can be estimated by

$$\begin{array}{l} {\hat{\sum }}_{\eta }=\frac{\overset{n}{\underset{i=1}{\sum }}\overset{n_{i}}{\underset{r=1}{\sum }}({\text{Z}}_{ir}-{\bar{\text{Z}}}_{i}){\left({\text{Z}}_{ir}-{\bar{\text{Z}}}_{i}\right)}^{⊤}}{\overset{n}{\underset{i=1}{\sum }}(n_{i}-1)}, \end{array}$$

where ${\bar{\text{Z}}}_{i}=\frac{1}{n_{i}}\overset{n_{i}}{\underset{r=1}{\sum }}{\text{Z}}_{ir}$. Alternatively, under the external validation study, in addition to the main study ℳ with the dataset $\left\{\{{\text{W}}_{i},{\text{Z}}_{i}\}:i\in ℳ\right\}$, the validation data 𝒱 with the availability of ${\text{X}}_{i}$ are collected, say $\left\{\{{\text{Z}}_{i},{\text{X}}_{i}\}:i\in 𝒱\right\}$. With the assumption of transportability and non‐overlapping of ℳ and 𝒱 imposed, ${\text{Z}}_{i}$ and ${\text{X}}_{i}$ can be characterized by (1) for i∈ℳ∪𝒱. Since ${\text{X}}_{i}$ and ${\text{Z}}_{i}$ are available for i∈𝒱, then we can respectively estimate $\sum_{X}≜\mathrm{var}(\text{X})$ and $\sum_{Z}$ by

$$\begin{array}{ll} {\hat{\sum }}_{X} & =\frac{1}{\left|𝒱\right|}\underset{i\in 𝒱}{\sum }\left(X_{i}-\bar{X}\right){\left(X_{i}-\bar{X}\right)}^{⊤}\;\;\text{and}\;\\ {\hat{\sum }}_{Z} & =\frac{1}{\left|𝒱\right|}\underset{i\in 𝒱}{\sum }\left(Z_{i}-\bar{Z}\right){\left(Z_{i}-\bar{Z}\right)}^{⊤},\; \end{array}$$

where $\bar{X}=\frac{1}{\left|𝒱\right|}\underset{i\in 𝒱}{\sum }X_{i}$ and $\bar{Z}=\frac{1}{\left|𝒱\right|}\underset{i\in 𝒱}{\sum }Z_{i}$. Consequently, based on the measurement error structure (1), $\sum_{\eta }$ can be estimated by ${\hat{\sum }}_{\eta }={\hat{\sum }}_{X}-{\hat{\sum }}_{Z}$.

### 2.2 Random Forest Estimation

Given the corrected dataset 𝒟 in Section 2.1, we implement the random forest (RF) method (e.g., [17], Chapter 15) to marginally characterize nonlinear effects of the covariates on each response. The RF method is a substantial modification of bagging that takes the average of a large collection of de‐correlated trees obtained by the B times resampling scheme. In general, the advantage of the RF method is to flexibly fit nonlinear relationship between the response and the covariates, and at the same time, determine the informative variables by the importance score. Theoretically, [18, 19] explored the consistency of the random forest method; and [20] examined the performance of variable selection for random forest when most predictive variables are noise.

Specifically, for the fixed jth response, let $D_{j}≜\{\{{\hat{Z}}_{i},{\hat{W}}_{ij}\}:i=1,\ldots ,n\}$ denote the “marginal” data with ${\hat{W}}_{ij}$ being the jth corrected response for the ith subject. In addition, let B denote the number of resampling procedures. For b=1,…,B, we take the sampling with replacement from ${𝒟}_{j}$ and obtain the resampled data, denoted as ${𝒟}_{jb}$. For the bth data ${𝒟}_{jb}$, a random forest tree, denoted as ${𝒯}_{jb}$, is grown by recursively selecting d corrected covariates at random from the full set at each node. Among these d variables, the algorithm identifies the optimal variable and corresponding split‐point to partition the data. At the same time, we adopt the variable importance plot in [17] (Section 15.3.2) to identify informative variables, which can be implemented by the function varImp in the R package caret. This splitting process continues until the minimum node size is reached. Repeating this procedure for b=1,…,B yields a class of B trees ${\left\{{𝒯}_{jb}\right\}}_{b=1}^{B}$ for the jth response with j=1,…,m. Consequently, the predicted outcomes for the jth response evaluated at the corrected covariate ${\hat{Z}}_{i}$ for all $i\in {𝒟}_{j}$ are given by

$${\tilde{W}}_{ij}≜\frac{1}{B}\overset{B}{\underset{b=1}{\sum }}{𝒯}_{jb}({\hat{Z}}_{i})$$

for j=1,…,m and $i\in {𝒟}_{j}$. Moreover, repeating the same procedure gives the whole fitted values.

Finally, we define the residual between the corrected response ${\hat{W}}_{ij}$ and the fitted value ${\tilde{W}}_{ij}$, which is given by

(4) $$r_{ij}={\hat{W}}_{ij}-{\tilde{W}}_{ij}$$

for i=1,…,n and j=1,…,m. We further define $r_{j}={\left(r_{1j},r_{2j},\ldots ,r_{nj}\right)}^{⊤}$ as the vector of the residual for the jth response for j=1,…,m.

### 2.3 Graphical Lasso and Network Detection

With the covariates ${\hat{\text{Z}}}_{i}$ accommodated, the residual (4) reflects the amount of variations of the jth response that has not been explained. In the presence of multivariate responses, the existence of pairwise dependence among responses is possible. It motivates us to adopt the residual to select other responses that provide higher marginal contributions associated with the jth residual for j=1,…,m, or equivalently, the stronger dependence associated with the jth response. On the other hand, note that the underlying relationship for any pair of responses is unknown and is possibly nonlinear. To detect potential responses in a flexible fashion, we employ the DC method (e.g., [21, 22]) that is a model free strategy to measure the correlation.

With a fixed j and $j^{\prime }$ with $j\neq j^{\prime }$, the DC is defined as

(5) $$\text{dcorr}(r_{j^{\prime }},r_{j})=\frac{\text{dcov}(r_{j^{\prime }},r_{j})}{\sqrt{\text{dcov}(r_{j^{\prime }},r_{j^{\prime }})\cdot \text{dcov}(r_{j},r_{j})}},$$

where

$$\begin{array}{l} \text{dcov}(\text{U},\text{V})=\int_{{\mathbb{R}}^{d_{U}+d_{V}}}{\left\|\phi_{\text{U},\text{V}}(r,s)-\phi_{\text{U}}(r)\phi_{\text{V}}(s)\right\|}^{2}w(r,s)drds \end{array}$$

is the distance covariance between $d_{U}$‐ and $d_{V}$‐dimensional random vectors U and V, where $\phi_{\text{U},\text{V}}(r,s)$ is the joint characteristic function of U and V, $\phi_{\text{U}}(r)$ and $\phi_{\text{V}}(s)$ are their respective marginal characteristic functions, and

$$\begin{array}{l} w(r,s)={\left\{c_{d_{U}}c_{d_{V}}{\left\|r\right\|}_{d_{U}}^{1+d_{U}}{\left\|s\right\|}_{d_{V}}^{1+d_{V}}\right\}}^{-1} \end{array}$$

with $c_{d}=\pi^{(1+d)/2}/\Gamma \{(1+d)/2\}$ and ${\left\|\text{a}\right\|}_{d}$ is the Euclidean norm of any vector $\text{a}\in {\mathbb{R}}^{d}$. A higher value of (5) reflects stronger correlation between $r_{j^{\prime }}$ and $r_{j}$, and thus, $r_{j^{\prime }}$ provides the most contribution to $r_{j}$, provided that $r_{j^{\prime }}$ has been explained by the informative covariates.

Following [22], dcov(U,V) can be re‐written as

(6) $$\begin{array}{l} \text{dcov}(\text{U},\text{V})=D_{1}+D_{2}-2D_{3}, \end{array}$$

where $D_{1}=E(\|\text{U}-\tilde{\text{U}}{\|}_{d_{U}}\|\text{V}-\tilde{\text{V}}{\|}_{d_{V}})$, $D_{2}=E(\|\text{U}-\tilde{\text{U}}{\|}_{d_{U}})\times E(\|\text{V}-\tilde{\text{V}}{\|}_{d_{V}})$, and $D_{3}=E\{E(\|\text{U}-\tilde{\text{U}}{\|}_{d_{U}}|\text{U})\times E(\|\text{V}-\tilde{\text{V}}{\|}_{d_{V}}|\text{V})\}$ with $\left(\tilde{\text{U}},\tilde{\text{V}}\right)$ being an independent copy of (U,V). With U and V in (6) replaced by $r_{j^{\prime }}$ and $r_{j}$, respectively, the estimator of (6), denoted $\hat{\text{dcov}}(r_{j^{\prime }},r_{j})$, is given by

(7) $$\hat{\text{dcov}}(r_{j^{\prime }},r_{j})={\hat{D}}_{1}+{\hat{D}}_{2}-2{\hat{D}}_{3},$$

where

$$\begin{array}{ll} {\hat{D}}_{1} & =\frac{1}{n^{2}}\overset{n}{\underset{i,k=1}{\sum }}\left|r_{ij^{\prime }}-r_{kj^{\prime }}\right|\cdot \left|r_{ij}-r_{kj}\right|,\\ {\hat{D}}_{2} & =\frac{1}{n^{2}}\overset{n}{\underset{i,k=1}{\sum }}\left|r_{ij^{\prime }}-r_{kj^{\prime }}\right|\times \frac{1}{n^{2}}\overset{n}{\underset{i,k=1}{\sum }}\left|r_{ij}-r_{kj}\right|, \end{array}$$

and

$${\hat{D}}_{3}=\frac{1}{n^{3}}\overset{n}{\underset{i,k,\ell =1}{\sum }}\left|r_{ij^{\prime }}-r_{\ell j^{\prime }}\right|\cdot \left|r_{kj}-r_{\ell j}\right|.$$

By the estimate (7), the DC (5) can then be estimated by ${\hat{\xi }}_{jj^{\prime }}≜\hat{\text{dcorr}}(r_{j^{\prime }},r_{j}).$ Since ${\hat{\xi }}_{jj^{\prime }}$ can be regarded as the “correlation” for nonlinear relationship between two variables, we further define the “correlation matrix” $\text{P}=[{\hat{\xi }}_{jj^{\prime }}]$.

To detect the network structure of the m responses, we adopt the glasso method (e.g., [7]) based on P to estimate the corresponding precision matrix of the response variables Y, denoted as $\Omega ≜[\omega_{jj^{\prime }}]$. Specifically, based on [7], we maximize the following optimization

(8) $$\hat{\Omega }≜\underset{\Omega ⪰\text{0}}{\text{argmax}}\left\{\log\det\Omega -\mathrm{tr}(\text{P}\Omega )-\lambda \|\Omega {\|}_{1}\right\},$$

where tr(A) is the trace of a matrix A, the notation “⪰0” indicates the positive semi‐definite precision matrix, λ is a tuning parameter, and $\left\|\Omega {\|}_{1}≜\underset{j\neq j^{\prime }}{\sum }|\omega_{jj^{\prime }}\right|$ is the $L_{1}$‐norm.

Unlike the conventional implementations that use the standard covariance matrices in (8), we adopt the matrix P. The main reason is that ${\hat{\xi }}_{jj^{\prime }}$ is specifically designed to detect dependent pairs of variables even in the presence of nonlinear relationships, thereby reflecting the underlying dependence structure. Additionally, ${\hat{\xi }}_{jj^{\prime }}$ is more sensitive in detecting dependent pairs of variables, whereas the empirical covariance is limited to capturing linear correlations. With penalty functions equipped in (8), zero‐valued entries in precision matrices can be also identified.

Theoretically, we discuss how this method works. Let $\xi_{jj^{\prime }}$ denote $\text{dcorr}(r_{j^{\prime }},r_{j})$ for simplicity of the notation. Following a similar derivation as in [22], we have that

(9) $$\begin{array}{l} P\left(\left|{\hat{\xi }}_{jj^{\prime }}-\xi_{jj^{\prime }}\right|\geq cn^{-\kappa }\right)\leq O\left\{\exp(-c_{1}n^{1-2(\kappa +\gamma )})+n\exp(-c_{2}n^{\gamma })\right\}, \end{array}$$

where $c,c_{1},c_{2}>0,\kappa \in [0,0.5)$, and γ∈(0,0.5−κ) are constants. It gives that

$$\begin{array}{ll} P\left(\max_{1\leq j,j^{\prime }\leq m}\left|{\hat{\xi }}_{jj^{\prime }}-\xi_{jj^{\prime }}\right|\geq cn^{-\kappa }\right) & \leq \frac{m(m-1)}{2}P\left(\left|{\hat{\xi }}_{jj^{\prime }}-\xi_{jj^{\prime }}\right|\geq cn^{-\kappa }\right)\\ & \leq O\left[\frac{m(m-1)}{2}\left\{\exp\left(-c_{1}n^{1-2(\kappa +\gamma )}\right)\right.\right.\\ & \;\left.\left.+n\exp(-c_{2}n^{\gamma })\right\}\right], \end{array}$$

implying that $\|\text{P}-{\text{P}}_{0}{\|}_{\max}≜\underset{1\leq j,j^{\prime }\leq m}{\max}\left|{\hat{\xi }}_{jj^{\prime }}-\xi_{jj^{\prime }}\right|$ is smaller than $cn^{-\kappa }$ with probability greater than $1-\frac{m(m-1)}{2}\left\{\exp(-c_{1}n^{1-2(\kappa +\gamma )})+n\exp(-c_{2}n^{\gamma })\right\}$, where ${\text{P}}_{0}=[\xi_{jj^{\prime }}]$. Consequently, provided the boundness of $\|\text{P}-{\text{P}}_{0}{\|}_{\max}$, the consistency and graph recovery of (8) can be ensured by following the proof of Theorem 4.3 in [23].

Moreover, in a special case that the relationship between $r_{j^{\prime }}$ and $r_{j}$ in (5) is linear, then $\xi_{jj^{\prime }}$ can be further written as (e.g., [22])

(10) $$\begin{array}{l} \xi_{jj^{\prime }}=\sqrt{\frac{\begin{array}{l} \rho_{jj^{\prime }}\sin^{-1}(\rho_{jj^{\prime }})+\sqrt{1-\rho_{jj^{\prime }}^{2}}-\rho_{jj^{\prime }}\sin^{-1}(\rho_{jj^{\prime }}⁄2)-\sqrt{4-\rho_{jj^{\prime }}^{2}}+1 \end{array}}{1+\pi ⁄3-\sqrt{3}}}, \end{array}$$

where $\rho_{jj^{\prime }}$ is the Pearson correlation coefficient. Consequently, we can estimate $\rho_{jj^{\prime }}$ by solving (10) with $\xi_{jj^{\prime }}$ replaced by ${\hat{\xi }}_{jj^{\prime }}$, and let ${\hat{\rho }}_{jj^{\prime }}$ denote the resulting estimator. Let ${\text{P}}_{0}^{\prime }=[\rho_{jj^{\prime }}]$ denote the matrix of the Pearson correlation coefficients and ${\text{P}}^{\prime }=[{\hat{\rho }}_{jj^{\prime }}]$ be the corresponding estimator of ${\text{P}}_{0}^{\prime }$. Let Ψ(ρ) be the function of ρ in the right‐hand side of (10) and let ξ denote the left‐hand side of (10) for the simplicity. Since, Ψ(ρ) is strictly increasing (e.g., [22]), the inverse of Ψ(ρ) exists, denoted $\Psi^{-1}(\xi )$, and is strictly increasing as well. In addition, due to the boundness of the DC ξ, $\Psi^{-1}(\xi )$ satisfies the Lipschitz condition, that is, $\left|{\hat{\rho }}_{jj^{\prime }}-\rho_{jj^{\prime }}|=|\Psi^{-1}({\hat{\xi }}_{jj^{\prime }})-\Psi^{-1}(\xi_{jj^{\prime }})|\leq \zeta |{\hat{\xi }}_{jj^{\prime }}-\xi_{jj^{\prime }}\right|$ for some constant ζ>0. Hence, by (9), we have that

$$\begin{array}{ll} P\left(\max_{1\leq j,j^{\prime }\leq m}|{\hat{\rho }}_{jj^{\prime }}-\rho_{jj^{\prime }}|\geq cn^{-\kappa }\right) & \leq \frac{m(m-1)}{2}P\left(|{\hat{\rho }}_{jj^{\prime }}-\rho_{jj^{\prime }}|\geq cn^{-\kappa }\right)\\ & \leq \frac{m(m-1)}{2}P\left(|{\hat{\xi }}_{jj^{\prime }}-\xi_{jj^{\prime }}|\geq \frac{c}{\zeta }n^{-\kappa }\right)\\ & \leq O\left[\frac{m(m-1)}{2}\left\{\exp\left(-c_{1}n^{1-2(\kappa +\gamma )}\right)\right.\right.\\ & \;\left.\left.+n\exp(-c_{2}n^{\gamma })\right\}\right], \end{array}$$

suggesting the consistency and graph recovery of (8) due to the probabilistic boundness of $\left\|{\text{P}}^{\prime }-{\text{P}}_{0}^{\prime }{\|}_{\max}≜\underset{1\leq j,j^{\prime }\leq m}{\max}|{\hat{\rho }}_{jj^{\prime }}-\rho_{jj^{\prime }}\right|$ (e.g., [23], Theorem 4.3). It also verifies the validity of ${\hat{\xi }}_{jj^{\prime }}$ in the linear relationship manner.

Finally, regarding the choice of the optimal λ, we employ the Bayesian information criterion (BIC). Specifically, let Λ denote a sequence of possible candidates of λ. For a fixed λ∈Λ, let $\hat{\Omega }(\lambda )$ denote the resulting estimator derived by (8). Then, the BIC is defined as

$$\begin{array}{l} \text{BIC}(\lambda )=-2\left\{\log\det\hat{\Omega }(\lambda )-\mathrm{tr}(\text{P}\hat{\Omega }(\lambda ))\right\}+h_{\lambda }\log(n), \end{array}$$

where $h_{\lambda }$ is the number of nonzero entries in $\hat{\Omega }(\lambda )$. The optimal tuning parameter can be determined by $\hat{\lambda }=\underset{\lambda \in \Lambda }{\mathrm{argmax}}\text{BIC}(\lambda )$, and thus, the resulting estimator of Ω is given by $\hat{\Omega }≜\hat{\Omega }(\hat{\lambda })$.

## 3 Simulation Studies

In this section, we conduct a series of simulation studies to assess the performance of the proposed method. Specifically, we consider the sample size and dimensions of the responses and covariates as (n,p,m)=(100,50,200),(400,20,50), (300,500,400), and (200,200,50), which include the dimensions are all smaller than the sample size, or the dimensions of covariates and/or responses are larger than the sample size. We generate the vector of covariates $X={\left(X_{1},X_{2},\ldots ,X_{p}\right)}^{⊤}$ by the standard multivariate normal distribution. After that, we consider the following two scenarios to generate the response $Y={\left(Y_{1},Y_{2},\ldots ,Y_{m}\right)}^{⊤}$:

*Scenario I* (Linear models): We generate Y by the multivariate linear models

$$\begin{array}{l} \text{Y}=\text{B}\text{X}+\epsilon , \end{array}$$

where B an m×p matrix with the left upper 6×6 block matrix being specified as

$$\begin{array}{l} {\text{B}}_{1}=\left(\begin{array}{cccccc} 1 & 0 & 0 & 0 & 0 & 0\\ 1 & 1 & 1 & 0 & 0 & 0\\ 1 & 1 & 0 & 1 & 0 & 0\\ 1 & 0 & 0 & 1 & 0 & 0\\ 0 & 0 & 0 & 0 & 0 & 0\\ 0 & 0 & 1 & 1 & 0 & 0 \end{array}\right) \end{array}$$

and other components being zero; ϵ follows the multivariate normal distribution with mean zero and the covariance matrix ∑.

*Scenario II* (Nonlinear models): In this scenario, we marginally generate Y as follows:

$$\begin{array}{ll} Y_{1} & =\sin(X_{1})+\epsilon_{1};\\ Y_{2} & =\sin(X_{1})+\cos(X_{2})+\sin(X_{3})+\epsilon_{2};\\ Y_{3} & =\varphi_{1}(Y_{1})+X_{1}+X_{2}^{2}+X_{4}^{2}+\epsilon_{3};\\ Y_{4} & =\sin(X_{1})+\cos(X_{4})+\epsilon_{4};\\ Y_{5} & =\epsilon_{5};\\ Y_{6} & =\varphi_{2}(Y_{4})+Y_{5}+\exp(X_{3})+X_{4}^{2}+\epsilon_{6}\\ Y_{j} & ∼N(0,1)\;\text{for}\;j=7,\ldots ,m, \end{array}$$

where $\epsilon_{j}∼N(0,1)$ for j=1,…,6, and $\varphi_{1}$ and $\varphi_{2}$ are two functions.

In Scenarios I and II, let ${𝒮}_{1}=\{1\}$, ${𝒮}_{2}=\{1,2,3\}$, ${𝒮}_{3}=\{1,2,4\}$, ${𝒮}_{4}=\{1,4\}$, and ${𝒮}_{6}=\{3,4\}$ denote the sets of informative covariate indices corresponding to responses $Y_{1},\cdots \;,Y_{4}$ and $Y_{6}$, respectively. These configurations indicate that only a subset of covariates is relevant for each response, reflecting the underlying sparsity structure in the model. On the other hand, we specify ∑ in Scenario I as the identity matrix and $\varphi_{1}=\varphi_{2}=0$ in Scenario II, so that the responses in the vector Y are fully independent of each other and there is no network structure. In contrast, we specify $\sum ={\left(\text{C}+\psi {\text{I}}_{m}\right)}^{-1}$ in Scenario I, where 


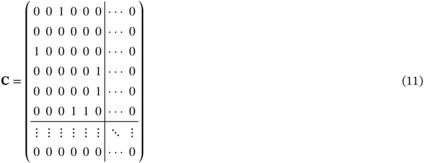


is an m×m precision matrix, ${\text{I}}_{m}$ is an m×m identity matrix, and ψ is the largest eigenvalue of (11); we specify $\varphi_{1}$ and $\varphi_{2}$ in Scenario II as

(12) $$\begin{array}{l} \varphi_{1}(x)=\exp(x)\;\text{and}\;\varphi_{2}(x)=\cos(x). \end{array}$$

Both (11) and (12) reflect the set of edges ℰ≜{(1,3),(5,6),(4,6)} in the true graph, and the resulting visualized network structure is displayed in Figure A.1 in the Supporting Information. In particular, (12) further reflects the nonlinear dependence between two responses. In the following implementations, we repeat 100 times for each setting. In addition, for the implementation of the proposed method, we employ a threshold of five for the importance scores, which serves as a well‐justified cutoff to distinguish informative variables from noise. Consequently, covariates with importance scores exceeding five for each response are retained as informative variables.

### 3.1 Simulation Results 1: Comparisons of Existing Methods

In this section, we compare with existing approaches, including the glasso [7], CLIME [8], Gaussian maximum likelihood (GML; [10]), and conditional likelihood estimation (CLE; [11]) methods, where the first two methods are simply implemented to detect the network structure of Y by using the existing R packages glasso and clime, respectively, and the last two methods impose the linear model structure for Y and X with the computational implementation reproduced by R programming language. All existing and proposed methods are implemented under R version 4.4.2 or higher. Provided that existing methods do not take measurement error into account, on the same line of precisely measured data $\left\{\{Y_{i},X_{i}\}:i=1,\ldots ,n\right\}$, we implement the proposed method in Section 2 with ${\hat{\text{Z}}}_{i}$ and ${\hat{\text{W}}}_{i}$ replaced by ${\text{X}}_{i}$ and ${\text{Y}}_{i}$, respectively, so that the advantage of the proposed estimation procedure can be emphasized.

Since the purpose is to select informative covariates and detect potential network structures of the responses, we primarily record specificity (SPE), sensitivity (SEN), and Matthews correlation coefficient (MCC) scores, which are respectively given by

$$\begin{array}{l} \text{SPE}=\frac{\text{TN}}{\text{TN}+\text{FP}},\;\;\text{SEN}=\frac{\text{TP}}{\text{TP}+\text{FN}}, \end{array}$$

and

$$\begin{array}{l} \text{MCC}=\frac{\text{TN}\times \text{TP}-\text{FN}\times \text{FP}}{\sqrt{\left(\text{TP}+\text{FN})\times (\text{TN}+\text{FP})\times (\text{TP}+\text{FP})\times (\text{TN}+\text{FN}\right)}}, \end{array}$$

where TP, TN, FP, and FN represent the numbers of true positives, true negatives, false positives, and false negatives between the truly informative covariates (or true network structure) and the selected informative covariates (or detected network structure) with “positive” being specified as informative covariates or pairs of connected responses.

Numerical results under Scenario II with the network structure in Figure A.1 in the Supporting Information are summarized in Table 1 and other results are placed in Tables A.1–A.3 in the Supporting Information. Moreover, to examine the stability of variable selection and network detection, we compute SPE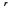, SEN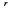, and MCC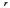 for each Monte Carlo repetition r=1,…,100. The empirical variances are then calculated based on these three sequences. For presentation purposes, these variances are scaled by a factor of 100 because their original values are extremely small.

**TABLE 1:** Simulation results under Scenario II and the network structure in Section 3.1. Values in parentheses are Monte Carlo variances scaled by a factor of 100.

		Variable selection			Network detection		
(n,p,m)	Methods	SPE	SEN	MCC	SPE	SEN	MCC
(100, 50, 200)	Glasso	—	—	—	0.096	1.000	0.023
		—	—	—	(0.070)	(0.001)	(0.118)
	CLIME	—	—	—	0.106	1.000	0.042
		—	—	—	(0.071)	(0.001)	(0.155)
	GML	0.775	0.909	0.054	0.974	1.000	0.399
		(0.001)	(0.405)	(0.500)	(0.061)	(0.138)	(0.266)
	CLE	0.745	0.890	0.034	0.908	1.000	0.350
		(0.010)	(0.423)	(0.520)	(0.082)	(0.144)	(0.272)
	Proposed	1.000	1.000	1.000	1.000	1.000	1.000
		(0.014)	(0.003)	(0.002)	(0.014)	(0.001)	(0.008)
(400, 50, 20)	Glasso	—	—	—	0.973	1.000	0.838
		—	—	—	(0.020)	(0.001)	(0.154)
	CLIME	—	—	—	0.950	1.000	0.840
		—	—	—	(0.017)	(0.001)	(0.163)
	GML	0.971	0.727	0.386	0.995	0.923	0.918
		(0.001)	(0.193)	(0.446)	(0.018)	(0.059)	(0.108)
	CLE	0.825	0.818	0.174	0.840	0.923	0.462
		(0.004)	(0.201)	(0.439)	(0.021)	(0.066)	(0.112)
	Proposed	0.998	1.000	0.919	1.000	1.000	1.000
		(0.007)	(0.001)	(0.054)	(0.001)	(0.005)	(0.003)
(300, 500, 400)	Glasso	—	—	—	0.948	1.000	0.717
		—	—	—	(0.023)	(0.001)	(0.166)
	CLIME	—	—	—	0.933	1.000	0.705
		—	—	—	(0.020)	(0.002)	(0.165)
	GML	—	—	—	—	—	—
	CLE	—	—	—	—	—	—
	Proposed	1.000	1.000	1.000	1.000	1.000	1.000
		(0.001)	(0.001)	(0.001)	(0.001)	(0.002)	(0.001)
(200, 200, 50)	Glasso	—	—	—	0.795	1.000	0.282
		—	—	—	(0.055)	(0.002)	(0.173)
	CLIME	—	—	—	0.776	1.000	0.410
		—	—	—	(0.059)	(0.002)	(0.168)
	GML	—	—	—	—	—	—
	CLE	—	—	—	—	—	—
	Proposed	1.000	1.000	1.000	0.998	1.000	0.944
		(0.001)	(0.001)	(0.001)	(0.003)	(0.001)	(0.020)

In general, we observe that the proposed method gives the highest values of SPE, SEN, and MCC over other existing methods for both variable selection and network detection regardless of the dimensions m and p, the model settings, and the existence of network structures in the responses. It implies that the importance plot enables us to detect informative covariates accurately, and the proposed modified glasso method is valid for detecting network structures precisely and verifies the theoretical findings in Section 2.3. On the other hand, without the incorporation of the covariates, the glasso and CLIME methods may have lower values of SPE and MCC, which indicate that some pairs may be falsely included. Regarding the results obtained by GML and CLE, the values of SPE, SEN, and MCC appear unsatisfactory in Scenario II, which is due to the fact that these methods are based on the linear structure and may not be useful to handle nonlinear models. In addition, under Scenario I, Tables A.1–A.3 in the Supporting Information reveal that the GML and CLE methods seem to be comparable to the proposed method. Moreover, it is also interesting to see that the GML and CLE methods may not produce the results when the dimensions m and p are large enough due to an unexpectedly long computation time. In contrast, we find that the proposed method is flexible and valid for handling complex settings, including the case where the dimensions are larger than the sample size. We also find that the proposed method gives the smallest empirical estimates of variances, which indicates that the proposed method gives stable variable selection and network detection.

### 3.2 Simulation Results 2: Examining the Impact of Measurement Errors

In this section, we focus on the estimation method proposed in Section 2 and examine the impact of ignorance or correction of measurement error. Based on the data Y and X generated in Scenarios I or II, we further generate W and Z by (1), where δ and η are independently generated from the multivariate normal distributions with mean zero and diagonal covariance matrices with common components $\sigma_{\delta }=0.2,0.5$ and $\sigma_{\eta }=0.2,0.5$ in $\sum_{\delta }$ and $\sum_{\eta }$, respectively.

We consider four settings for measurement error correction of responses and/or covariates, including error in both responses and covariates (EE), error in responses and correction for covariates (EC), correction for responses and error in covariates (CE), and correction for both responses and covariates (CC). The first three approaches are regarded as naive methods without measurement error correction, and the CC method is the proposed method.

In addition to the evaluation criteria SPE, SEN, and MCC used in Section 3.1 to assess detection accuracy, we further examine the impact of measurement error by computing the biases of the estimated linear/nonlinear functions. Specifically, let $f_{jk}(x)$ denote the true regression function and ${\hat{f}}_{jk}(x)$ the corresponding estimate obtained by the random forest method for j∈{1,2,3,4,6} and $k\in {𝒮}_{j}$. For each pair $(j,k)\in ℐ≜\left\{(j^{\prime },k^{\prime }):j^{\prime }\in \{1,2,3,4,6\}\;\text{and}\;k^{\prime }\in {𝒮}_{j^{\prime }}\right\}$, we define the individual bias as

$$\begin{array}{l} \|\Delta {ϒ}_{jk}{\|}_{1}≜\overset{n}{\underset{i=1}{\sum }}|{\hat{f}}_{jk}({\hat{Z}}_{ik})-f_{jk}({\hat{Z}}_{ik})|, \end{array}$$

where ${\hat{Z}}_{ik}$ is the k the corrected covariate for the ith subject; it can be replaced by $Z_{ik}$ is measurement error correction is ignored. To summarize the overall estimation accuracy, we define the average bias across all relevant functions as

(13) $$\begin{array}{l} \|\Delta ϒ{\|}_{1}≜\frac{1}{\left|ℐ\right|}\underset{(j,k)\in ℐ}{\sum }\|\Delta {ϒ}_{jk}{\|}_{1}. \end{array}$$

This metric integrates the biases from multiple function estimates into a single summary measure. Furthermore, for each Monte Carlo repetition r=1,…,100, we compute (13), denoted as $\|\Delta ϒ{\|}_{1r}$, and utilize the resulting sequence $\left\{\|\Delta \Upsilon {\|}_{1r}:r=1,\ldots ,100\right\}$ to calculate the empirical variance scaled by a factor of 100. Additionally, we evaluate the empirical variances for SPE, SEN, and MCC, as described in Section 3.1.

Numerical results for Scenario II with the network structure in Figure A.1 in the Supporting Information are placed in Table 2, and other results are summarized in Tables A.4–A.6 in the Supporting Information. First, from Scenarios I and II, the proposed method (CC) consistently yields the smallest estimation bias, measured by (13), outperforming all alternative approaches. This result supports the validity of the regression calibration strategy in effectively addressing measurement error. Comparing Scenarios I and II, it is not surprising that the biases are generally smaller under the linear setting. This is likely because regression calibration is more naturally aligned with linear structures and may be less effective in fully correcting nonlinear distortions. In contrast, methods that fail to correct for measurement error in responses, covariates, or both, such as EE, EC, and CE, exhibit substantially larger biases. Among them, the EE approach performs the worst, indicating the critical importance of jointly correcting measurement error in both components of the model.

**TABLE 2:** Simulation results under Scenario II and the network structure in Section 3.2. Values in parentheses are Monte Carlo variances scaled by a factor of 100.

			Variable selection				Network detection		
(n,p,m)	Error	Correction	$\|\Delta ϒ{\|}_{1}$	SPE	SEN	MCC	SPE	SEN	MCC
(100, 50, 200)	(0.2, 0.2)	EE	10.948	0.906	0.909	0.578	0.990	0.980	0.900
			(1.978)	(0.001)	(0.035)	(0.015)	(0.001)	(0.001)	(0.001)
		EC	8.633	0.980	0.909	0.823	0.999	0.981	0.926
			(1.822)	(0.001)	(0.090)	(0.015)	(0.001)	(0.001)	(0.001)
		CE	10.838	0.999	1.000	0.677	0.999	0.981	0.900
			(1.945)	(0.001)	(0.072)	(0.010)	(0.001)	(0.001)	(0.001)
		CC	6.610	1.000	0.909	0.944	1.000	0.981	0.985
			(1.233)	(0.001)	(0.098)	(0.014)	(0.001)	(0.001)	(0.001)
	(0.5, 0.5)	EE	13.890	0.557	0.818	0.190	0.895	0.990	0.203
			(2.033)	(0.001)	(0.058)	(0.019)	(0.001)	(0.002)	(0.001)
		EC	8.770	0.785	0.818	0.350	0.894	0.990	0.202
			(1.911)	(0.001)	(0.056)	(0.021)	(0.001)	(0.002)	(0.001)
		CE	13.676	0.999	0.909	0.691	0.996	0.990	0.746
			(2.021)	(0.001)	(0.047)	(0.021)	(0.001)	(0.002)	(0.001)
		CC	6.179	1.000	0.981	0.987	0.999	0.990	0.913
			(1.450)	(0.001)	(0.039)	(0.027)	(0.001)	(0.002)	(0.001)
(400, 50, 20)	(0.2, 0.2)	EE	15.208	0.765	1.000	0.428	0.824	1.000	0.482
			(1.747)	(0.005)	(0.078)	(0.036)	(0.001)	(0.030)	(0.016)
		EC	12.153	0.718	1.000	0.386	0.807	1.000	0.463
			(1.520)	(0.005)	(0.063)	(0.032)	(0.001)	(0.030)	(0.022)
		CE	15.044	0.664	1.000	0.346	0.850	1.000	0.519
			(1.739)	(0.006)	(0.039)	(0.036)	(0.001)	(0.001)	(0.014)
		CC	9.080	1.000	0.909	0.944	1.000	1.000	1.000
			(1.226)	(0.010)	(0.039)	(0.050)	(0.001)	(0.030)	(0.027)
	(0.5, 0.5)	EE	22.088	0.718	0.909	0.339	0.823	1.000	0.496
			(1.905)	(0.009)	(0.063)	(0.026)	(0.001)	(0.030)	(0.027)
		EC	12.147	0.685	0.818	0.267	0.856	1.000	0.527
			(1.613)	(0.001)	(0.053)	(0.087)	(0.001)	(0.056)	(0.058)
		CE	21.770	0.691	1.000	0.365	0.856	1.000	0.527
			(1.808)	(0.004)	(0.051)	(0.018)	(0.001)	(0.056)	(0.032)
		CC	7.789	0.918	0.909	0.957	0.989	1.000	0.926
			(1.330)	(0.005)	(0.043)	(0.071)	(0.001)	(0.056)	(0.058)
(300, 500, 400)	(0.2, 0.2)	EE	3.700	1.000	1.000	1.000	0.992	1.000	0.477
			(2.019)	(0.024)	(0.047)	(0.022)	(0.004)	(0.001)	(0.050)
		EC	3.546	1.000	1.000	1.000	0.992	1.000	0.477
			(1.843)	(0.001)	(0.024)	(0.047)	(0.002)	(0.001)	(0.094)
		CE	3.553	1.000	1.000	1.000	0.992	1.000	0.481
			(2.014)	(0.001)	(0.041)	(0.020)	(0.001)	(0.002)	(0.038)
		CC	3.150	1.000	1.000	1.000	1.000	1.000	1.000
			(1.466)	(0.001)	(0.028)	(0.024)	(0.001)	(0.002)	(0.067)
	(0.5, 0.5)	EE	4.210	0.996	1.000	0.117	0.996	1.000	0.642
			(2.107)	(0.001)	(0.053)	(0.027)	(0.002)	(0.018)	(0.017)
		EC	3.774	0.996	1.000	0.118	0.997	1.000	0.658
			(2.046)	(0.001)	(0.088)	(0.0035)	(0.003)	(0.014)	(0.018)
		CE	3.952	0.996	1.000	0.118	0.996	1.000	0.636
			(2.089)	(0.001)	(0.072)	(0.026)	(0.001)	(0.012)	(0.012)
		CC	3.107	1.000	1.000	1.000	1.000	1.000	1.000
			(1.690)	(0.001)	(0.087)	(0.044)	(0.002)	(0.014)	(0.014)
(200, 200, 50)	(0.2, 0.2)	EE	13.124	0.705	1.000	0.466	0.990	1.000	0.950
			(1.938)	(0.001)	(0.079)	(0.025)	(0.002)	(0.001)	(0.013)
		EC	10.425	0.774	1.000	0.512	0.998	1.000	0.965
			(1.772)	(0.001)	(0.092)	(0.028)	(0.001)	(0.001)	(0.078)
		CE	13.051	0.803	1.000	0.645	0.998	1.000	0.965
			(1.906)	(0.001)	(0.085)	(0.040)	(0.002)	(0.001)	(0.021)
		CC	7.435	1.000	1.000	1.000	0.999	1.000	0.982
			(1.530)	(0.001)	(0.099)	(0.040)	(0.001)	(0.001)	(0.013)
	(0.5, 0.5)	EE	15.279	1.000	0.909	0.953	0.647	1.000	0.199
			(2.049)	(0.001)	(0.082)	(0.028)	(0.001)	(0.006)	(0.091)
		EC	9.168	1.000	0.909	0.953	0.639	1.000	0.195
			(1.901)	(0.001)	(0.060)	(0.018)	(0.001)	(0.022)	(0.076)
		CE	14.963	1.000	0.909	0.909	0.659	1.000	0.204
			(1.965)	(0.001)	(0.021)	(0.034)	(0.003)	(0.006)	(0.023)
		CC	6.942	1.000	1.000	0.957	1.000	1.000	1.000
			(1.608)	(0.001)	(0.005)	(0.046)	(0.001)	(0.022)	(0.090)

Next, when measurement errors in both responses and covariates are properly corrected, the proposed method achieves the highest values of SPE, SEN, and MCC among all approaches. This demonstrates that the integration of random forest and regression calibration effectively identifies informative covariates, regardless of whether the underlying model structure is linear or nonlinear. In contrast, failing to account for measurement error can adversely affect variable selection, either by missing relevant covariates or by incorrectly including noninformative ones, leading to substantially lower SPE, SEN, and MCC values.

In terms of network detection performance, the proposed method accurately recovers the estimated network to the underlying network structure, achieving values of SPE, SEN, and MCC close to one regardless of the regression model type or the relationship between dimensionality and sample size. This result supports the theoretical findings presented in Section 2.3. In contrast, methods that do not account for measurement error, such as EE, EC, and CE, exhibit notably lower values of SPE and MCC. These reductions indicate that spurious edges may be incorrectly included, preventing the accurate recovery of the true network structure. This highlights the substantial impact of measurement error on network estimation accuracy.

Finally, we observe that the CC approach produces the smallest empirical variances for (13), SPE, SEN, and MCC, indicating that its performance is relatively stable. In contrast, the remaining three (EE, EC, and CE) approaches yield relatively larger empirical variances, suggesting that their instability may be attributed to the presence of measurement errors.

### 3.3 Simulation Results 3: Estimation With the Availability of Auxiliary Information

In this section, we examine the estimation in the presence of auxiliary information. We follow the same settings as in Sections 3 and 3.2 to generate the data, except that two covariance matrices $\sum_{\delta }$ and $\sum_{\eta }$ are respectively specified as

*Setting A*: $\left[\sigma_{\delta }^{1+|i-j|}\right]$ and $\left[\sigma_{\eta }^{1+|k-l|}\right]$ for i,j=1,⋯,m and k,l=1,⋯,p;

*Setting B*: $\left[0.8\times \sigma_{\delta }\right]+(0.2\times \sigma_{\delta }){\text{I}}_{m}$ and $\left[0.8\times \sigma_{\eta }\right]+(0.2\times \sigma_{\eta }){\text{I}}_{p}$,

where $\sigma_{\delta }$ and $\sigma_{\eta }$ are 0.2 or 0.5. Noting that matrices in Setting A exhibit a decaying covariance structure, while those in Setting B feature common off‐diagonal entries. In addition, we consider repeated measurements and validation data as auxiliary information. Specifically, we generate five repeated measurements ${\text{Z}}_{ir}$ and ${\text{W}}_{ir}$ by (1) with the same sample size as defined in Section 3.2. On the other hand, for the validation sample, we additionally generate ${\text{W}}_{i}$, ${\text{Y}}_{i}$, ${\text{Z}}_{i}$, and ${\text{X}}_{i}$ by (1) with sample size 2n. Based on auxiliary information, we adopt the methods in Section 2.1 to estimate $\sum_{\delta }$ and $\sum_{\eta }$ defined in Settings A and B, and then plug in the estimators to the estimation procedure in Sections 2.2 and 2.3.

The simulation results under Settings A and B are respectively presented in Tables A.7–A.10 and Tables A.11–A.14 in the Supporting Information, where “RM” denotes results obtained using repeated measurements, and “VD” refers to those based on validation samples. Consistent with the findings in Section 3.2, the proposed method effectively estimates unknown functions of covariates, identifies informative covariates, and accurately recovers the underlying network structure, provided that the error covariance matrices $\sum_{\delta }$ and $\sum_{\eta }$ are estimated using either repeated measurements or validation data. The results, including biases, SPE, SEN, MCC, and their empirical variances, are comparable across both Settings A and B, as well as both types of auxiliary information. This suggests that the proposed method is stable to the choice of estimation strategy for the measurement error covariance. Furthermore, the method maintains satisfactory performance even when $\sum_{\delta }$ and $\sum_{\eta }$ are not diagonal, demonstrating its flexibility in more general settings.

## 4 GBM Data Analysis

In this section, we apply the proposed method to analyze the GBM dataset. As introduced in Section 1, our objective is twofold: to model the relationship between microRNA and gene expressions, and to uncover the underlying network structure among microRNAs using informative gene expression covariates. Following the preprocessing steps described in [11], we remove samples with missing values and retain the top 500 gene expressions and top 20 microRNAs with the largest median absolute deviations. The resulting dataset contains n=196 samples, with p=500 gene expression variables and m=20 microRNA responses.

This dataset was previously analyzed by [10, 11], both of whom assumed a linear relationship between gene expression profiles and microRNA expression levels. In contrast, our approach relaxes this assumption and offers a more flexible framework that accounts for potential nonlinearities. Additionally, we address the challenge of measurement error, which can affect both gene and microRNA expression measurements. Let W and Z denote the observed microRNA and gene expression data, respectively. Since the covariance matrices $\sum_{\delta }$ and $\sum_{\eta }$ are unknown, we perform sensitivity analyses and specify $\sum_{\delta }$ and $\sum_{\eta }$ by the following two scenarios:

*Scenario I* (Diagonal matrices): We assume diagonal structures with shared values $\sigma_{\delta }=\sigma_{\eta }=0,0.2$, or 0.5.

*Scenario II* (Non‐diagonal matrices): Following Chen and Yi [15], we specify $\sum_{\delta }$ and $\sum_{\eta }$ as

$$\begin{array}{l} \sum_{\delta }=(1-R_{W}){\hat{\sum }}_{\text{W}}\;\;\text{and}\;\;\sum_{\eta }=(1-R_{Z}){\hat{\sum }}_{\text{Z}}, \end{array}$$

where ${\hat{\sum }}_{\text{W}}$ and ${\hat{\sum }}_{\text{Z}}$ are empirical estimates of two covariance matrices of microRNA responses and gene expression variables, respectively, and $R_{W}$ and $R_{Z}$ are the reliability ratios with common values R=0.5,0.8, or 1.

Noting that $\sigma_{\delta }=\sigma_{\eta }=0$ and $R_{W}=R_{Z}=1$ correspond to the naive model without measurement error correction.

**FIGURE 1:**
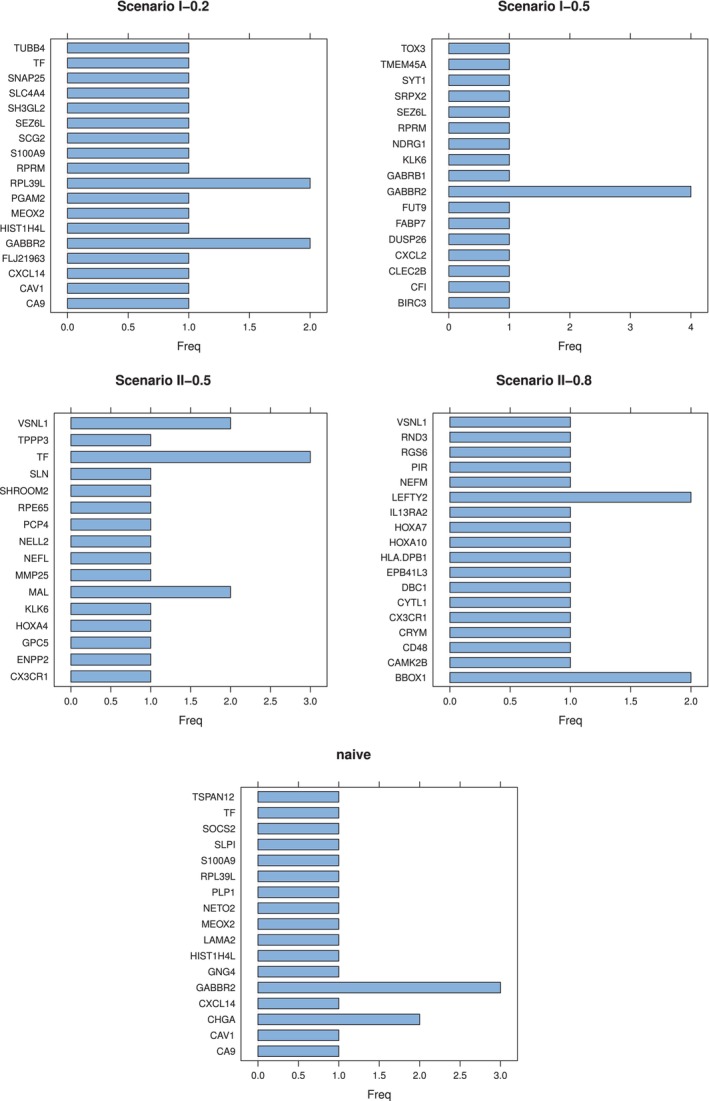
Real data analysis results: Selection of informative gene expressions. The caption I‐y indicates Scenario I with $\sigma_{\eta }=\sigma_{\delta }=y$ for y=0.2 or 0.5; the caption II‐r indicates Scenario II with $R_{W}=R_{Z}=r$ for r=0.5 or 0.8; the caption naive reflects $\sigma_{\eta }=\sigma_{\delta }=0$.

We first apply the random forest method described in Section 2.2 to regress each microRNA on the gene expressions and compute variable importance scores, where $\sum_{\delta }$ and $\sum_{\eta }$ are specified as in Scenarios I and II. We retain the covariates with importance scores greater than five for each response and treat them as informative variables. The numbers of selected gene expressions under various specifications in Scenarios I and II are summarized in Table 3. We observe that $R_{W}=R_{Z}=0.5$ under Scenario II selects the most covariates among all settings, while $\sigma_{\delta }=\sigma_{\eta }=0.5$ Scenario I suggests the smallest value of the selected covariates. In the same line of ignorance of the measurement error, the naive method and [11] suggest a similar number of selected gene expressions. To see which gene expression incurs the most significant impact on microRNA, we summarize the frequency of the covariate with the highest importance score among 20 microRNA responses in Figure 1. We observe that the gene expression GABBR2 is frequently selected by the naive method and under Scenario I regardless of the choices of $\sigma_{\eta }$ and $\sigma_{\delta }$. In contrast, under Scenario II, it is interesting to see that different specifications of $R_{W}$ and $R_{Z}$ give various frequency of variable selection. That is, two gene expressions TF, VSNL1, and MAL are selected when $R_{W}=R_{Z}=0.5$, while two gene expressions LEFTY2 and BBOX1 are selected when $R_{W}=R_{Z}=0.8$. The main reason is that $\sum_{\delta }$ and $\sum_{\eta }$ share the common values under Scenario I, which may provide stable selection. In contrast, components in Scenario II are different and dependent on empirical estimates ${\hat{\sum }}_{\text{W}}$ and ${\hat{\sum }}_{\text{Z}}$, which may affect the selection of gene expressions.

**TABLE 3:** Real data analysis results: Predicted square errors (PSE) and number of selected covariates (VS). In the “Scenario” column, the label I‐y indicates Scenario I with $\sigma_{\eta }=\sigma_{\delta }=y$ for y=0.2 or 0.5; the label II‐r indicates Scenario II with $R_{W}=R_{Z}=r$ for r=0.5 or 0.8; the label naive reflects $\sigma_{\eta }=\sigma_{\delta }=0$.

Scenario	PSE	VS
I‐0.2	0.752	73
I‐0.5	0.666	50
II‐0.5	0.502	104
II‐0.8	0.781	53
Naive	0.991	75

In addition, to see the performance of estimation, we compute the predicted values ${\hat{W}}_{ij}$ and then determine the predicted square errors (PSE) based on (4), that is, $\frac{1}{nm}\overset{n}{\underset{i=1}{\sum }}\overset{m}{\underset{j=1}{\sum }}r_{ij}^{2}$. The results are placed in Table 3. In general, regardless of the specification of $\sum_{\delta }$ and $\sum_{\eta }$ from two scenarios, the proposed method generally outperforms the naive method with smaller values of PSE. More specifically, we find that specifying $R_{W}=R_{Z}=0.5$ in Scenario II gives the smallest value of PSE. In the same line of the minor error effects, Scenario I ($\sigma_{\eta }=\sigma_{\delta }=0.2$) produces comparable PSE to that determined by Scenario II ($R_{W}=R_{Z}=0.8$). On the other hand, when measurement error is not corrected, the value of PSE determined by the naive method is smaller than that obtained by other existing methods reported in [11]. This finding reflects that the underlying relationship between microRNA and gene expressions might be nonlinear.

**FIGURE 2:**
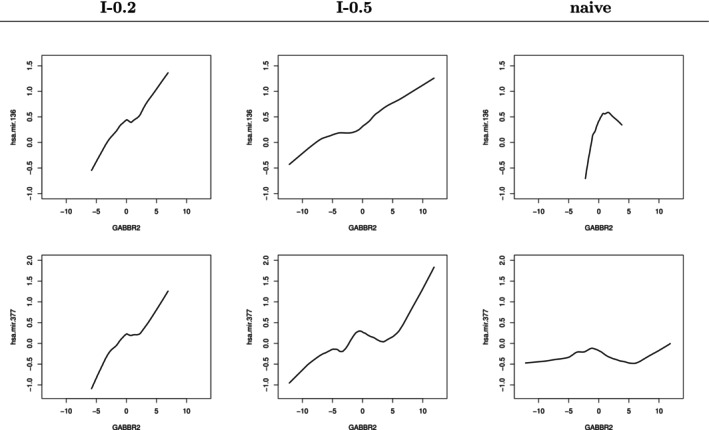
Real data analysis results: Model fitting of the GABBR2 and three microRNAs (hsa.mir.136, hsa.mir.376, and hsa.mir.377) commonly selected across different scenarios. The caption I‐y indicates Scenario I with $\sigma_{\eta }=\sigma_{\delta }=y$ for y=0.2 or 0.5; the caption naive reflects $\sigma_{\eta }=\sigma_{\delta }=0$.

To further explore how gene expressions affect microRNA and the corresponding relationship, we adopt the random forest method to fit the highest frequent gene expressions in Figure 1 and their associated microRNAs. Due to the limited space in the main text, we display GABBR2 and corresponding common microRNAs in Figure 2 since it is commonly selected by Scenario I and the naive method, and other results corresponding to unique pairs of microRNAs and gene expressions determined by individual setting are displayed in Figures B.1–B.5 in the Supporting Information. In general, all figures reveal that selected gene expressions have nonlinear relationships with their corresponding microRNAs. Moreover, it is also interesting to observe from Figure 2 that the relationships between GABBR2 and hsa.mir.136as well as hsa.mir.377 show increasing pattern and have positive correlation when measurement error effects are taken into account ($\sigma_{\eta }=\sigma_{\delta }=0.2$ or 0.5), while the patterns become different if measurement error effects are ignored by the naive method. This finding provides an evidence of impacts caused by measurement errors. In addition, the ranges of fitted microRNAs shown in Figure 2 are different with the changes of $\sigma_{\eta }$ and $\sigma_{\delta }$, which reflect that magnitudes of measurement errors may affect the fitness of regression models.

**FIGURE 3:**
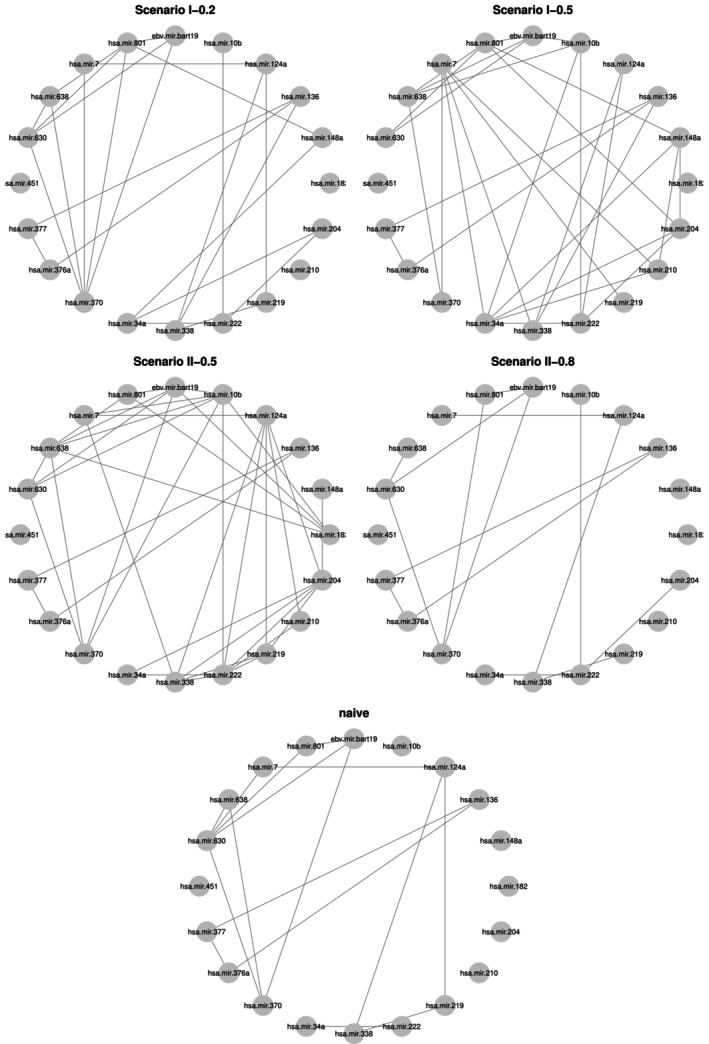
Real data analysis results: estimated network structures. The caption I‐y indicates Scenario I with $\sigma_{\eta }=\sigma_{\delta }=y$ for y=0.2 or 0.5; the caption II‐r indicates Scenario II with $R_{W}=R_{Z}=r$ for r=0.5 or 0.8; the caption naive reflects $\sigma_{\eta }=\sigma_{\delta }=0$.

Given the informative gene expressions, we adopt the estimation method in Section 2.3 to determine the estimated network structures under Scenarios I and II. The corresponding results are displayed in Figure 3. Compared with existing methods under linear models (e.g., [10, 11]), the network structures obtained by the proposed and naive methods are relatively sparse. Among the comparisons of the proposed method, we find that some pairs are commonly detected regardless of scenarios, such as a pair (ebv.mir.bart19, hsa.mir.801) and a triangle (hsa.mir.136, hsa.mir.376a, hsa.mir.377) that is also detected by [11]. In addition, we observe that some pairs are uniquely selected under some specific scenarios. For example, pairs (ebv.mir.124a, hsa.mir.210) and (ebv.mir.204,hsa.mir.338) are selected when $R_{W}=R_{Z}=0.8$ in Scenario II is considered and two pairs (ebv.mir.148a, hsa.mir.210) and (ebv.mir.148a, hsa.mir.801) are detected under Scenario I.

To further see the relationships among pairs of microRNAs, we display the corresponding diagrams in Figures B.6–B.9 of the Supporting Information. We first notice from Figure B.6 in Supporting Information that pairs in a triangle (hsa.mir.136, hsa.mir.376a, hsa.mir.377) reflect positive and linear relationships, except for pairs (hsa.mir.136, hsa.mir.376a) and (hsa.mir.376a, hsa.mir.377) detected by $\sigma_{\eta }=\sigma_{\delta }=0.5$ in Scenario I. These findings may explain why [11] also captured those pairs under linear models. On the other hand, pairs (hsa.mir.148, hsa.mir.210) and (hsa.mir.148a, hsa.mir.801) in Figures B.8 and B.9 of the Supporting Information show slightly nonlinear relationships. This finding may explain why they are not identified by [11]. In addition, while Supporting Information Figure B.7 shows strictly decreasing patterns for pairs (hsa.mir.124a, hsa.mir.210) and (hsa.mir.204, hsa.mir.338), they are detected when measurement errors are corrected, which is also a reason that they are not detected by the naive method and [11]. In summary, an insight of this finding includes the importance of measurement error correction and validity of the proposed method for detecting nonlinear effects of two microRNAs.

## 5 Summary

In this paper, we study the modeling for multivariate responses and covariates. Our goals include the selection of informative covariates and detection of the network structure for responses. While several methods have been available to address those two issues, several critical assumptions are implicitly imposed in existing methods, including (i) linear relationship and parametric form between responses and covariates and (ii) both responses and covariates are precisely measured. Those settings could be restrictive and unrealistic in applications, especially for bioinformatics studies. To tackle those challenges, we introduce a model‐free strategy to model the responses and the covariates. Our method is flexible to handle fully unknown relationship between the responses and covariates, rather than requiring the working model or prior knowledge of regression models. The regression calibration method is valid for dealing with measurement error, and the extension of the graphical lasso method enables us to detect the network structure when the covariates are equipped. While the current development focuses on continuous random variables due to the motivated data, our method can be naturally extended to handle the multivariate responses with mixed distributions, provided that the measurement error and residuals can be well defined.

## Funding

This work was supported by the National Science and Technology Council (Grant No. 114‐2118‐M‐004‐010‐MY3).

## Conflicts of Interest

The author declares no conflicts of interest.

## Supporting information




**Figure A.1:** The graphical structure in simulation studies.
**Table A.1:** Simulation results under Scenario II and the independence structure in Section 3.1. Values in parentheses are Monte Carlo variances scaled by a factor of 100.
**Table A.2:** Simulation results under Scenario I and the network structure in Section 3.1. Values in parentheses are Monte Carlo variances scaled by a factor of 100.
**Table A.3:** Simulation results under Scenario I and the independence structure in Section 3.1. Values in parentheses are Monte Carlo variances scaled by a factor of 100.
**Table A.4:** Simulation results under Scenario II and the independence structure in Section 3.2. Values in parentheses are Monte Carlo variances scaled by a factor of 100.
**Table A.5:** Simulation results under Scenario I and the network structure in Section 3.2. Values in parentheses are Monte Carlo variances scaled by a factor of 100.
**Table A.6:** Simulation results under Scenario I and the independence structure in Section 3.2. Values in parentheses are Monte Carlo variances scaled by a factor of 100.
**Table A.7:** Simulation results under Scenario II, Setting A, and the network structure in Section 3.3. Values in parentheses are Monte Carlo variances scaled by a factor of 100.
**Table A.8:** Simulation results under Scenario II, Setting A, and the independence structure in Section 3.3. Values in parentheses are Monte Carlo variances scaled by a factor of 100.
**Table A.9:** Simulation results under Scenario I, Setting A, and the network structure in Section 3.3. Values in parentheses are Monte Carlo variances scaled by a factor of 100.
**Table A.10:** Simulation results under Scenario I, Setting A, and the independence structure in Section 3.3. Values in parentheses are Monte Carlo variances scaled by a factor of 100.
**Table A.11:** Simulation results under Scenario II, Setting B, and the network structure in Section 3.3. Values in parentheses are Monte Carlo variances scaled by a factor of 100.
**Table A.12:** Simulation results under Scenario II, Setting B, and the independence structure in Section 3.3. Values in parentheses are Monte Carlo variances scaled by a factor of 100.
**Table A.13:** Simulation results under Scenario I, Setting B, and the network structure in Section 3.3. Values in parentheses are Monte Carlo variances scaled by a factor of 100.
**Table A.14:** Simulation results under Scenario I, Setting B, and the independence structure in Section 3.3. Values in parentheses are Monte Carlo variances scaled by a factor of 100.
**Figure B.1:** Real data analysis results: Model fitting of a gene RPL39L and two microRNAs (hsa.mir.370 and hsa.mir.630) under $\sigma_{\delta }=\sigma_{\eta }=0.2$ in Scenario I.
**Figure B.2:** Real data analysis results: Model fitting of a gene GABBR2 and three microRNAs (hsa.mir.451, hsa.mir.630, and hsa.mir.801) $\sigma_{\delta }=\sigma_{\eta }=0.5$ in Scenario I.
**Figure B.3:** Real data analysis results: Model fitting of three genes (MAL, TF, and VSNL1) and several microRNAs (hsa.mir.7, hsa.mir.204, hsa.mir.136, hsa.mir.376a, hsa.mir.377, hsa.mir.124a, and hsa.mir.222) under $R_{W}=R_{Z}=0.5$ in Scenario II.
**Figure B.4:** Real data analysis results: Model fitting of two genes (BBOX1 and LEFTY2) and four microRNAs (hsa.mir.204, hsa.mir.222, hsa.mir.219, and hsa.mir.338) under $R_{W}=R_{Z}=0.8$ in Scenario II.
**Figure B.5:** Real data analysis results: Model fitting of two genes (GABBR2 and CHGA) and three microRNAs (hsa.mir.376a, hsa.mir.7, and hsa.mir.124a) determined by the naive method.
**Figure B.6:** Real data analysis results: Model fitting of the pairs of microRNAs commonly selected across different scenarios. The caption I‐y indicates Scenario I with $\sigma_{\eta }=\sigma_{\delta }=y$ for y=0.2 or 0.5; the caption II‐r indicates Scenario II with $R_{W}=R_{Z}=r$ for r=0.5 or 0.8; the caption naive reflects $\sigma_{\eta }=\sigma_{\delta }=0$.
**Figure B.7:** Real data analysis results: Model fitting of two pairs (hsa.mir.124a, hsa.mir.210) and (hsa.mir.204, hsa.mir.338) uniquely selected under $R_{W}=R_{Z}=0.8$ in Scenario II.
**Figure B.8:** Real data analysis results: Model fitting of the pair (hsa.mir.630, hsa.mir.7) uniquely selected under $\sigma_{\delta }=\sigma_{\eta }=0.5$ in Scenario I.
**Figure B.9:** Real data analysis results: Model fitting of a pair (hsa.mir.148a, hsa.mir.801) selected under $\sigma_{\delta }=\sigma_{\eta }=0.2$ (left penal) and 0.5 (right panel) in Scenario I.
The supporting information contains additional results for simulation studies and real data analysis. R programming code for simulation studies and real data analysis are placed at the GitHub https://github.com/lchen723/RF_Graph.

## Data Availability

The data that supports the findings of this study are available in the Supporting Information of this article.
