# Supplementary material for: Predictor‐Assisted Nonparametric Graphical Models With Multivariate Error‐Prone Data
Source: Stat Med. 2026 Jul 7;45(15-17):e70658. doi: 10.1002/sim.70658 (PMC13339170; doi:10.1002/sim.70658)
Supplement: Supplementary file 1 — Figure A.1: The graphical structure in simulation studies. Table A.1: Simulation results under Scenario II and the independence structure in Section 3.1. Values in parentheses are Monte Carlo variances scaled by a factor of 100. Table A.2: Simulation results under Scenario I and the network structure in Section 3.1. Values in parentheses are Monte Carlo variances scaled by a factor of 100. Table A.3: Simulation results under Scenario I and the independence structure in Section 3.1. Values in parentheses are Monte Carlo variances scaled by a factor of 100. Table A.4: Simulation results under Scenario II and the independence structure in Section 3.2. Values in parentheses are Monte Carlo variances scaled by a factor of 100. Table A.5: Simulation results under Scenario I and the network structure in Section 3.2. Values in parentheses are Monte Carlo variances scaled by a factor of 100. Table A.6: Simulation results under Scenario I and the independence structure in Section 3.2. Values in parentheses are Monte Carlo variances scaled by a factor of 100. Table A.7: Simulation results under Scenario II, Setting A, and the network structure in Section 3.3. Values in parentheses are Monte Carlo variances scaled by a factor of 100. Table A.8: Simulation results under Scenario II, Setting A, and the independence structure in Section 3.3. Values in parentheses are Monte Carlo variances scaled by a factor of 100. Table A.9: Simulation results under Scenario I, Setting A, and the network structure in Section 3.3. Values in parentheses are Monte Carlo variances scaled by a factor of 100. Table A.10: Simulation results under Scenario I, Setting A, and the independence structure in Section 3.3. Values in parentheses are Monte Carlo variances scaled by a factor of 100. Table A.11: Simulation results under Scenario II, Setting B, and the network structure in Section 3.3. Values in parentheses are Monte Carlo variances scaled by a factor of 100. Table A.12: Simulation results under Sce [file SIM-45-0-s001.pdf]

Supporting Information of "Predictor-Assisted Nonparametric Graphical Models with Multivariate Error-Prone Data"

Li-Pang Chen
Department of Statistics, National Chengchi University
Email: lchen723@nccu.edu.tw

Appendix A Additional Simulation Results

In this appendix, we summarize additional simulation results from the main text, including nonlinear models without network structure, linear models with/without network structure. Specifically,

- Figure A.1 displays the visualized network structure in the simulation setting.
- Tables A.1-A.3 summarize the comparisons with other existing methods under the true responses and covariates in Section 3.1;
- Tables A.4-A.6 summarize the comparisons of measurement error correction for responses, covariates, or both in Section 3.2;
- Tables A.7-A.10 summarize the results of measurement error correction by using auxiliary information, including repeated measurements and validation data for Setting A in the main text in Section 3.3.
- Tables A.11-A.14 summarize the results of measurement error correction by using auxiliary information, including repeated measurements and validation data for Setting B in the main text in Section 3.3.

In general, the results are consistent with the findings in the main text. The proposed method is generally better than other existing methods and is valid for handling measurement error. It implies that the proposed method is flexible to handle linear models and accurately detects the network structure.

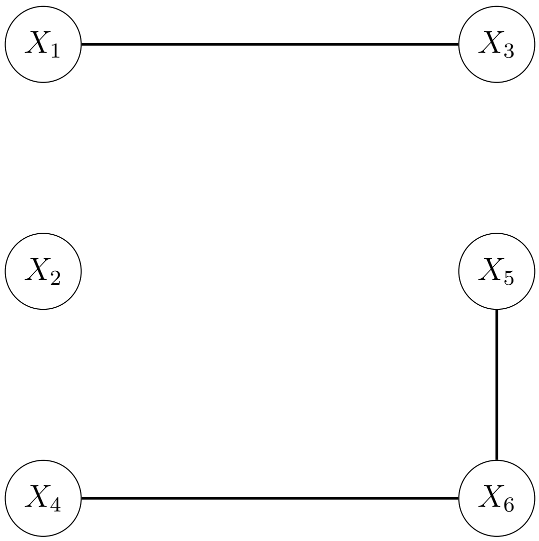

Figure A.1: The graphical structure in simulation studies

Table A.1: Simulation results under Scenario II and the independence structure in Section 3.1. Values in parentheses are Monte Carlo variances scaled by a factor of 100.

(n,p,m)	Methods	Variable Selection			Network Detection		
		SPE	SEN	MCC	SPE	SEN	MCC
(100,50,200)	glasso	–	–	–	0.093	1.000	0.023
		–	–	–	(0.237)	(0.026)	(0.099)
	CLIME	–	–	–	0.050	1.000	0.020
		–	–	–	(0.225)	(0.022)	(0.106)
	GML	0.770	1.000	0.061	0.974	1.000	0.399
		(0.147)	(0.001)	(0.256)	(0.097)	(0.028)	(0.138)
	CLE	0.711	1.000	0.049	0.893	1.000	0.320
		(0.155)	(0.001)	(0.278)	(0.104)	(0.021)	(0.145)
	Proposed	1.000	1.000	1.000	1.000	1.000	0.985
		(0.001)	(0.004)	(0.005)	(0.018)	(0.029)	(0.034)
(400,50,20)	glasso	–	–	–	0.973	1.000	0.838
		–	–	–	(0.010)	(0.001)	(0.116)
	CLIME	–	–	–	0.950	0.980	0.821
		–	–	–	(0.011)	(0.007)	(0.123)
	GML	0.970	0.727	0.380	0.984	1.000	0.870
		(0.010)	(0.083)	(0.095)	(0.158)	(0.023)	(0.243)
	CLE	0.834	0.909	0.204	0.845	1.000	0.462
		(0.008)	(0.090)	(0.087)	(0.163)	(0.049)	(0.280)
	Proposed	1.000	1.000	1.000	1.000	1.000	1.000
		(0.008)	(0.001)	(0.006)	(0.001)	(0.001)	(0.004)
(300,500,400)	glasso	–	–	–	–	–	–
	CLIME	–	–	–	–	–	–
	GML	–	–	–	–	–	–
	CLE	–	–	–	–	–	–
	Proposed	1.000	1.000	1.000	1.000	1.000	1.000
		(0.001)	(0.001)	(0.001)	(0.001)	(0.001)	(0.001)
(200,200,50)	glasso	–	–	–	0.773	1.000	0.266
		–	–	–	(0.156)	(0.001)	(0.230)
	CLIME	–	–	–	0.775	0.996	0.252
		–	–	–	(0.155)	(0.012)	(0.242)
	GML	–	–	–	–	–	–
	CLE	–	–	–	–	–	–
	Proposed	1.000	1.000	1.000	0.999	1.000	0.980
		(0.001)	(0.001)	(0.001)	(0.003)	(0.001)	(0.011)

Table A.2: Simulation results under Scenario I and the network structure in Section 3.1. Values in parentheses are Monte Carlo variances scaled by a factor of 100.

(n, p, m)	Methods	Variable Selection			Network Detection		
		SPE	SEN	MCC	SPE	SEN	MCC
(100,50,200)	glasso	–	–	–	0.097	1.000	0.023
		–	–	–	(0.191)	(0.006)	(0.149)
	CLIME	–	–	–	0.083	1.000	0.025
		–	–	–	(0.211)	(0.010)	(0.154)
	GML	0.913	1.000	0.107	0.998	1.000	0.854
		(0.023)	(0.001)	(0.120)	(0.001)	(0.004)	(0.454)
	CLE	0.908	1.000	0.097	0.985	1.000	0.833
		(0.020)	(0.001)	(0.135)	(0.005)	(0.003)	(0.466)
	Proposed	1.000	1.000	1.000	1.000	0.981	0.990
		(0.003)	(0.001)	(0.002)	(0.002)	(0.024)	(0.027)
(400,50,20)	glasso	–	–	–	0.947	0.923	0.685
		–	–	–	(0.002)	(0.098)	(0.055)
	CLIME	–	–	–	0.850	1.000	0.630
		–	–	–	(0.010)	(0.007)	(0.074)
	GML	1.000	1.000	1.000	1.000	0.769	0.870
		(0.004)	(0.003)	(0.003)	(0.001)	(0.040)	(0.025)
	CLE	0.984	1.000	0.633	0.911	1.000	0.581
		(0.007)	(0.002)	(0.033)	(0.012)	(0.037)	(0.046)
	Proposed	1.000	1.000	1.000	1.000	0.923	0.958
		(0.003)	(0.001)	(0.002)	(0.002)	(0.067)	(0.016)
(300,500,400)	glasso	–	–	–	0.797	0.990	0.703
		–	–	–	(0.160)	(0.015)	(0.183)
	CLIME	–	–	–	0.744	0.905	0.680
		–	–	–	(0.195)	(0.022)	(0.209)
	GML	–	–	–	–	–	–
	CLE	–	–	–	–	–	–
	Proposed	1.000	1.000	1.000	1.000	0.995	0.995
		(0.001)	(0.001)	(0.001)	(0.001)	(0.004)	(0.003)
(200,200,50)	glasso	–	–	–	0.818	1.000	0.302
		–	–	–	(0.144)	(0.001)	(0.245)
	CLIME	–	–	–	0.750	1.000	0.290
		–	–	–	(0.160)	(0.001)	(0.273)
	GML	–	–	–	–	–	–
	CLE	–	–	–	–	–	–
	Proposed	1.000	1.000	1.000	0.998	0.964	0.930
		(0.001)	(0.001)	(0.001)	(0.002)	(0.010)	(0.014)

Table A.3: Simulation results under Scenario I and the independence structure in Section 3.1. Values in parentheses are Monte Carlo variances scaled by a factor of 100.

(n, p, m)	Methods	Variable Selection			Network Detection		
		SPE	SEN	MCC	SPE	SEN	MCC
(100,50,200)	glasso	–	–	–	0.726	1.000	0.342
		–	–	–	(0.022)	(0.001)	(0.186)
	CLIME	–	–	–	0.078	1.000	0.022
		–	–	–	(0.068)	(0.002)	(0.194)
	GML	0.769	1.000	0.060	0.974	1.000	0.395
		(0.043)	(0.002)	(0.055)	(0.022)	(0.003)	(0.063)
	CLE	0.774	1.000	0.075	0.935	1.000	0.388
		(0.039)	(0.001)	(0.059)	(0.028)	(0.005)	(0.071)
	Proposed	1.000	1.000	1.000	1.000	1.000	1.000
		(0.002)	(0.001)	(0.001)	(0.008)	(0.001)	(0.004)
(400,50,20)	glasso	–	–	–	0.937	1.000	0.653
		–	–	–	(0.001)	(0.001)	(0.020)
	CLIME	–	–	–	0.850	1.000	0.620
		–	–	–	(0.026)	(0.002)	(0.033)
	GML	1.000	1.000	1.000	1.000	1.000	1.000
		(0.005)	(0.005)	(0.003)	(0.003)	(0.002)	(0.002)
	CLE	0.941	1.000	0.387	0.963	1.000	0.753
		(0.020)	(0.004)	(0.057)	(0.023)	(0.002)	(0.044)
	Proposed	1.000	1.000	1.000	1.000	1.000	1.000
		(0.003)	(0.001)	(0.005)	(0.002)	(0.001)	(0.005)
(300,500,400)	glasso	–	–	–	0.898	0.895	0.720
		–	–	–	(0.145)	(0.093)	(0.178)
	CLIME	–	–	–	0.705	1.000	0.630
		–	–	–	(0.181)	(0.002)	(0.196)
	GML	–	–	–	–	–	–
	CLE	–	–	–	–	–	–
	Proposed	1.000	1.000	1.000	1.000	1.000	1.000
		(0.001)	(0.001)	(0.001)	(0.001)	(0.001)	(0.001)
(200,200,50)	glasso	–	–	–	0.798	1.000	0.270
		–	–	–	(0.166)	(0.001)	(0.234)
	CLIME	–	–	–	0.650	1.000	0.192
		–	–	–	(0.189)	(0.002)	(0.241)
	GML	–	–	–	–	–	–
	CLE	–	–	–	–	–	–
	Proposed	1.000	1.000	1.000	1.000	1.000	1.000
		(0.001)	(0.001)	(0.001)	(0.001)	(0.001)	(0.001)

Table A.4: Simulation results under Scenario II and the independence structure in Section 3.2. Values in parentheses are Monte Carlo variances scaled by a factor of 100.

(n, p, m)	Error	Correction	Variable Selection				Network Detection		
			$\|\Delta\boldsymbol{\Upsilon}\|_1$	SPE	SEN	MCC	SPE	SEN	MCC
(100,50,200)	(0.2,0.2)	EE	8.809	0.906	0.909	0.578	0.999	0.981	0.897
			(1.894)	(0.001)	(0.033)	(0.022)	(0.001)	(0.004)	(0.001)
		EC	6.557	0.973	0.909	0.790	0.999	0.990	0.913
			(1.633)	(0.001)	(0.039)	(0.019)	(0.001)	(0.005)	(0.001)
		CE	8.718	0.999	1.000	0.781	0.999	0.981	0.890
			(1.845)	(0.001)	(0.070)	(0.017)	(0.001)	(0.005)	(0.001)
		CC	3.935	1.000	0.909	0.944	1.000	0.971	0.985
			(1.334)	(0.001)	(0.092)	(0.016)	(0.001)	(0.003)	(0.001)
	(0.5,0.5)	EE	11.871	0.604	0.909	0.262	0.893	1.000	0.200
			(1.956)	(0.001)	(0.051)	(0.035)	(0.001)	(0.002)	(0.001)
		EC	5.974	0.812	0.818	0.378	0.893	1.000	0.200
			(1.740)	(0.001)	(0.068)	(0.038)	(0.001)	(0.003)	(0.001)
		CE	11.684	0.999	0.818	0.592	0.892	1.000	0.199
			(1.932)	(0.001)	(0.078)	(0.029)	(0.001)	(0.001)	(0.002)
		CC	4.311	1.000	0.918	0.887	1.000	1.000	0.995
			(1.411)	(0.001)	(0.081)	(0.040)	(0.001)	(0.003)	(0.001)
(400,50,20)	(0.2,0.2)	EE	14.811	0.638	1.000	0.328	0.858	1.000	0.482
			(1.645)	(0.009)	(0.029)	(0.088)	(0.056)	(0.001)	(0.028)
		EC	10.300	0.691	0.909	0.318	0.816	1.000	0.426
			(1.453)	(0.005)	(0.017)	(0.048)	(0.001)	(0.056)	(0.073)
		CE	14.696	0.705	0.909	0.328	0.863	1.000	0.490
			(1.633)	(0.005)	(0.013)	(0.061)	(0.056)	(0.001)	(0-28)
		CC	7.650	0.980	0.909	0.889	1.000	1.000	1.000
			(1.266)	(0.008)	(0.039)	(0.056)	(0.001)	(0.056)	(0.077)
	(0.5,0.5)	EE	18.576	0.597	1.000	0.304	0.853	1.000	0.474
			(1.846)	(0.006)	(0.035)	(0.067)	(0.001)	(0.080)	(0.089)
		EC	8.410	0.752	0.909	0.367	0.847	1.000	0.466
			(1.560)	(0.014)	(0.064)	(0.049)	(0.010)	(0.002)	(0.018)
		CE	18.309	0.698	1.000	0.370	0.826	1.000	0.438
			(1.820)	(0.005)	(0.065)	(0.011)	(0.001)	(0.080)	(0.093)
		CC	6.299	1.000	0.909	0.944	1.000	1.000	1.000
			(1.345)	(0.010)	(0.056)	(0.044)	(0.002)	(0.031)	(0.033)

(300,500,400)	(0.2,0.2)	EE	11.444	1.000	0.818	0.905	0.994	1.000	0.534
			(2.300)	(0.001)	(0.309)	(0.013)	(0.001)	(0.001)	(0.045)
		EC	8.166	1.000	0.818	0.905	0.993	1.000	0.526
			(1.845)	(0.001)	(0.787)	(0.048)	(0.005)	(0.001)	(0.165)
		CE	11.306	1.000	0.818	0.905	0.993	1.000	0.524
			(2.031)	(0.001)	(0.364)	(0.017)	(0.001)	(0.001)	(0.059)
		CC	6.385	1.000	0.909	0.953	1.000	1.000	1.000
			(1.530)	(0.001)	(0.066)	(0.013)	(0.005)	(0.001)	(0.002)
	(0.5,0.5)	EE	16.487	0.924	1.000	0.026	0.997	1.000	0.669
			(2.637)	(0.034)	(0.001)	(0.071)	(0.003)	(0.001)	(0.120)
		EC	7.677	0.923	1.000	0.026	0.997	1.000	0.659
			(2.120)	(0.033)	(0.001)	(0.075)	(0.002)	(0.001)	(0.083)
		CE	16.232	0.924	1.000	0.026	0.996	1.000	0.644
			(2.450)	(0.026)	(0.001)	(0.061)	(0.002)	(0.001)	(0.101)
		CC	5.707	1.000	1.000	1.000	1.000	1.000	1.000
			(1.744)	(0.001)	(0.002)	(0.001)	(0.002)	(0.001)	(0.003)
(200,200,50)	(0.2,0.2)	EE	11.748	1.000	1.000	0.909	0.659	1.000	0.193
			(2.203)	(0.001)	(0.079)	(0.027)	(0.002)	(0.029)	(0.024)
		EC	8.236	1.000	1.000	1.000	0.680	1.000	0.202
			(1.893)	(0.001)	(0.038)	(0.040)	(0.002)	(0.045)	(0.044)
		CE	11.633	1.000	1.000	1.000	0.651	1.000	0.190
			(2.119)	(0.001)	(0.091)	(0.024)	(0.003)	(0.031)	(0.061)
		CC	6.352	1.000	1.000	1.000	1.000	1.000	1.000
			(1.530)	(0.001)	(0.076)	(0.021)	(0.002)	(0.031)	(0.042)
	(0.5,0.5)	EE	15.043	1.000	0.909	0.870	0.667	1.000	0.196
			(2.589)	(0.001)	(0.090)	(0.028)	(0.001)	(0.031)	(0.098)
		EC	7.206	1.000	0.909	0.909	0.680	1.000	0.202
			(2.034)	(0.001)	(0.065)	(0.018)	(0.002)	(0.044)	(0.047)
		CE	14.834	1.000	0.909	0.909	0.669	1.000	0.197
			(2.203)	(0.001)	(0.029)	(0.033)	(0.002)	(0.029)	(0.055)
		CC	5.066	1.000	0.909	0.953	1.000	1.000	1.000
			(1.833)	(0.001)	(0.029)	(0.041)	(0.002)	(0.044)	(0.058)

Table A.5: Simulation results under Scenario I and the network structure in Section 3.2. Values in parentheses are Monte Carlo variances scaled by a factor of 100.

(n,p,m)	Error	Correction	Variable Selection				Network Detection		
			$\|\Delta\boldsymbol{\Upsilon}\|_1$	SPE	SEN	MCC	SPE	SEN	MCC
(100,50,200)	(0.2,0.2)	EE	3.133	0.998	1.000	0.605	0.049	1.000	0.016
			(1.743)	(0.001)	(0.002)	(0.002)	(0.001)	(0.003)	(0.003)
		EC	2.981	0.997	1.000	0.499	0.051	1.000	0.017
			(1.533)	(0.002)	(0.078)	(0.001)	(0.00)	(0.010)	(0.012)
		CE	3.037	0.998	1.000	0.577	0.049	1.000	0.016
			(1.708)	(0.001)	(0.041)	(0.001)	(0.002)	(0.003)	(0.002)
		CC	2.542	1.000	1.000	1.000	0.917	1.000	0.886
			(1.330)	(0.001)	(0.078)	(0.002)	(0.002)	(0.002)	(0.005)
	(0.5,0.5)	EE	3.587	0.960	1.000	0.691	0.049	1.000	0.016
			(1.833)	(0.001)	(0.039)	(0.003)	(0.001)	(0.003)	(0.001)
		EC	3.252	0.996	1.000	0.488	0.048	1.000	0.016
			(1.616)	(0.001)	(0.034)	(0.033)	(0.001)	(0.002)	(0.001)
		CE	3.324	0.997	1.000	0.537	0.051	1.000	0.017
			(1.802)	(0.001)	(0.051)	(0.004)	(0.001)	(0.001)	(0.002)
		CC	2.461	0.999	1.000	0.938	0.917	1.000	0.886
			(1.445)	(0.00)	(0.024)	(0.033)	(0.001)	(0.001)	(0.002)
(400,50,20)	(0.2,0.2)	EE	3.656	0.999	1.000	0.957	0.917	0.923	0.792
			(1.770)	(0.006)	(0.001)	(0.044)	(0.002)	(0.056)	(0.095)
		EC	3.470	0.999	1.000	0.957	0.963	1.000	0.791
			(1.659)	(0.011)	(0.002)	(0.049)	(0.001)	(0.002)	(0.047)
		CE	3.531	1.000	1.000	1.000	0.973	0.923	0.792
			(1.740)	(0.008)	(0.002)	(0.054)	(0.002)	(0.056)	(0.050)
		CC	3.061	1.000	1.000	1.000	0.973	1.000	0.886
			(1.356)	(0.011)	(0.002)	(0.050)	(0.001)	(0.003)	(0.057)
	(0.5,0.5)	EE	4.253	0.993	1.000	0.779	0.963	0.846	0.697
			(1.848)	(0.007)	(0.001)	(0.045)	(0.003)	(0.030)	(0.045)
		EC	3.801	0.993	1.000	0.957	0.947	0.846	0.637
			(1.690)	(0.010)	(0.002)	(0.019)	(0.005)	(0.056)	(0.018)
		CE	3.967	0.999	1.000	0.957	0.963	0.846	0.697
			(1.796)	(0.008)	(0.005)	(0.057)	(0.002)	(0.030)	(0.015)
		CC	3.131	1.000	1.000	1.000	1.000	1.000	0.906
			(1.403)	(0.014)	(0.003)	(0.028)	(0.05)	(0.056)	(0.059)

(300,500,400)	(0.2,0.2)	EE	3.558	0.924	1.000	0.026	0.997	0.990	0.649
			(1.863)	(0.010)	(0.010)	(0.001)	(0.001)	(0.018)	(0.064)
		EC	3.426	0.922	1.000	0.026	0.996	0.990	0.641
			(1.731)	(0.010)	(0.001)	(0.011)	(0.001)	(0.001)	(0.043)
		CE	3.430	0.924	1.000	0.026	0.997	0.990	0.661
			(1.850)	(0.002)	(0.001)	(0.001)	(0.003)	(0.018)	(0.048)
		CC	2.958	1.000	1.000	1.000	1.000	1.000	1.000
			(1.449)	(0.001)	(0.001)	(0.001)	(0.001)	(0.001)	(0.003)
	(0.5,0.5)	EE	4.183	0.924	1.000	0.026	0.996	1.000	0.636
			(1.902)	(0.001)	(0.005)	(0.001)	(0.018)	(0.001)	(0.080)
		EC	3.718	0.924	1.000	0.026	0.996	1.000	0.643
			(1.793)	(0.001)	(0.001)	(0.001)	(0.029)	(0.001)	(0.103)
		CE	3.905	0.924	1.000	0.026	0.997	1.000	0.645
			(1.892)	(0.011)	(0.001)	(0.001)	(0.018)	(0.001)	(0.086)
		CC	2.862	1.000	1.000	1.000	1.000	1.000	1.000
			(1.488)	(0.001)	(0.001)	(0.002)	(0.001)	(0.002)	(0.002)
(200,200,50)	(0.2,0.2)	EE	3.403	1.000	1.000	0.957	0.782	1.000	0.272
			(1.903)	(0.001)	(0.002)	(0.003)	(0.002)	(0.016)	(0.098)
		EC	3.223	1.000	1.000	0.957	0.783	1.000	0.274
			(1.745)	(0.001)	(0.002)	(0.003)	(0.001)	(0.001)	(0.086)
		CE	3.266	1.000	1.000	0.957	0.796	1.000	0.284
			(1.896)	(0.002)	(0.002)	(0.002)	(0.002)	(0.012)	(0.013)
		CC	2.910	1.000	1.000	1.000	1.000	1.000	1.000
			(1.453)	(0.001)	(0.001)	(0.003)	(0.001)	(0.006)	(0.085)
	(0.5,0.5)	EE	3.867	1.000	1.000	1.000	0.657	0.964	0.192
			(1.966)	(0.002)	(0.041)	(0.001)	(0.002)	(0.012)	(0.016)
		EC	3.487	1.000	1.000	1.000	0.656	0.964	0.192
			(1.784)	(0.001)	(0.078)	(0.005)	(0.002)	(0.033)	(0.022)
		CE	3.607	1.000	1.000	1.000	0.668	0.964	0.197
			(1.930)	(0.001)	(0.002)	(0.001)	(0.001)	(0.006)	(0.090)
		CC	2.817	1.000	1.000	1.000	1.000	1.000	1.000
			(1.506)	(0.001)	(0.011)	(0.004)	(0.002)	(0.027)	(0.028)

Table A.6: Simulation results under Scenario I and the independence structure in Section 3.2. Values in parentheses are Monte Carlo variances scaled by a factor of 100.

(n,p,m)	Error	Correction	Variable Selection				Network Detection		
			$\|\Delta\boldsymbol{\Upsilon}\|_1$	SPE	SEN	MCC	SPE	SEN	MCC
(100,50,200)	(0.2,0.2)	EE	3.358	0.839	1.000	0.513	0.995	0.990	0.711
			(1.711)	(0.021)	(0.001)	(0.002)	(0.001)	(0.002)	(0.001)
		EC	3.223	0.973	1.000	0.845	0.995	0.990	0.707
			(1.588)	(0.001)	(0.002)	(0.002)	(0.001)	(0.003)	(0.002)
		CE	3.236	0.999	1.000	0.723	0.995	0.990	0.697
			(1.695)	(0.001)	(0.001)	(0.002)	(0.002)	(0.003)	(0.002)
		CC	2.857	1.000	1.000	1.000	1.000	1.000	1.000
			(1.256)	(0.001)	(0.002)	(0.002)	(0.001)	(0.002)	(0.002)
	(0.5,0.5)	EE	3.714	0.658	1.000	0.342	0.892	1.000	0.199
			(1.789)	(0.001)	(0.011)	(0.002)	(0.001)	(0.002)	(0.002)
		EC	3.355	0.906	1.000	0.631	0.893	1.000	0.200
			(1.609)	(0.001)	(0.052)	(0.019)	(0.002)	(0.002)	(0.001)
		CE	3.473	0.999	1.000	0.741	0.894	1.000	0.201
			(1.758)	(0.001)	(0.027)	(0.005)	(0.001)	(0.002)	(0.002)
		CC	2.599	0.980	1.000	0.948	0.999	1.000	0.945
			(1.311)	(0.001)	(0.024)	(0.013)	(0.002)	(0.002)	(0.001)
(400,50,20)	(0.2,0.2)	EE	3.765	0.503	1.000	0.255	0.732	1.000	0.346
			(1.766)	(0.007)	(0.002)	(0.053)	(0.002)	(0.002)	(0.097)
		EC	3.628	0.792	1.000	0.455	0.700	1.000	0.323
			(1.645)	(0.007)	(0.003)	(0.031)	(0.002)	(0.004)	(0.019)
		CE	3.630	0.517	1.000	0.262	0.716	1.000	0.334
			(1.739)	(0.006)	(0.003)	(0.039)	(0.002)	(0.002)	(0.010)
		CC	3.220	0.980	1.000	0.948	1.000	1.000	1.000
			(1.345)	(0.006)	(0.001)	(0.028)	(0.002)	(0.002)	(0.018)
	(0.5,0.5)	EE	4.288	0.564	1.000	0.286	0.689	1.000	0.316
			(1.946)	(0.007)	(0.002)	(0.036)	(0.005)	(0.002)	(0.021)
		EC	3.856	0.597	1.000	0.304	0.753	1.000	0.363
			(1.728)	(0.010)	(0.002)	(0.017)	(0.010)	(0.003)	(0.029)
		CE	3.990	0.617	1.000	0.316	0.711	1.000	0.331
			(1.886)	(0.007)	(0.003)	(0.039)	(0.005)	(0.001)	(0.021)
		CC	3.205	1.000	1.000	1.000	0.989	1.000	0.908
			(1.397)	(0.012)	(0.003)	(0.022)	(0.007)	(0.005)	(0.019)

(300,500,400)	(0.2,0.2)	EE	15.043	1.000	0.818	0.905	0.989	1.000	0.436
			(1.814)	(0.001)	(0.011)	(0.010)	(0.005)	(0.001)	(0.100)
		EC	11.852	1.000	0.818	0.905	0.990	1.000	0.451
			(1.533)	(0.001)	(0.018)	(0.011)	(0.005)	(0.001)	(0.110)
		CE	14.923	1.000	0.818	0.905	0.990	1.000	0.450
			(1.793)	(0.001)	(0.018)	(0.010)	(0.006)	(0.001)	(0.103)
		CC	8.916	1.000	1.000	1.000	1.000	1.000	1.000
			(1.345)	(0.001)	(0.001)	(0.002)	(0.001)	(0.001)	(0.006)
	(0.5,0.5)	EE	18.565	0.544	1.000	0.275	0.699	1.000	0.401
			(1.946)	(0.008)	(0.001)	(0.012)	(0.001)	(0.001)	(0.078)
		EC	10.731	0.557	1.000	0.282	0.730	1.000	0.435
			(1.596)	(0.010)	(0.001)	(0.013)	(0.003)	(0.001)	(0.072)
		CE	18.189	0.537	1.000	0.272	0.753	1.000	0.454
			(1.860)	(0.011)	(0.001)	(0.012)	(0.004)	(0.001)	(0.096)
		CC	8.880	1.000	1.000	1.000	1.000	1.000	1.000
			(1.426)	(0.001)	(0.002)	(0.001)	(0.001)	(0.001)	(0.004)
(200,200,50)	(0.2,0.2)	EE	3.546	1.000	1.000	0.957	0.656	1.000	0.192
			(1.743)	(0.001)	(0.002)	(0.001)	(0.001)	(0.003)	(0.072)
		EC	3.398	1.000	1.000	0.920	0.671	1.000	0.198
			(1.520)	(0.001)	(0.001)	(0.002)	(0.002)	(0.002)	(0.018)
		CE	3.419	1.000	1.000	0.920	0.637	1.000	0.184
			(1.719)	(0.001)	(0.001)	(0.002)	(0.001)	(0.002)	(0.079)
		CC	2.995	1.000	1.000	1.000	1.000	1.000	1.000
			(1.358)	(0.001)	(0.001)	(0.001)	(0.002)	(0.001)	(0.017)
	(0.5,0.5)	EE	3.985	0.564	1.000	0.266	0.660	1.000	0.193
			(1.892)	(0.001)	(0.001)	(0.003)	(0.003)	(0.001)	(0.017)
		EC	3.607	0.590	1.000	0.298	0.659	1.000	0.193
			(1.633)	(0.001)	(0.037)	(0.011)	(0.002)	(0.001)	(0.098)
		CE	3.732	0.608	1.000	0.330	0.669	1.000	0.197
			(1.813)	(0.001)	(0.001)	(0.002)	(0.002)	(0.001)	(0.014)
		CC	2.963	1.000	1.000	0.957	1.000	1.000	1.000
			(1.469)	(0.001)	(0.039)	(0.012)	(0.001)	(0.002)	(0.039)

Table A.7: Simulation results under Scenario II, Setting A, and the network structure in Section 3.3. Values in parentheses are Monte Carlo variances scaled by a factor of 100.

(n,p,m)	Error	Information	Variable Selection				Network Detection		
			$\|\Delta\mathbf{\Upsilon}\|_1$	SPE	SEN	MCC	SPE	SEN	MCC
(100,50,200)	(0.2,0.2)	RM	5.462	1.000	0.909	0.953	1.000	1.000	1.000
			(1.894)	(0.001)	(0.019)	(0.027)	(0.001)	(0.001)	(0.001)
		VD	5.523	1.000	0.927	0.953	1.000	1.000	1.000
			(1.815)	(0.001)	(0.082)	(0.027)	(0.001)	(0.001)	(0.001)
	(0.5,0.5)	RM	5.505	1.000	0.918	0.904	0.997	1.000	0.904
			(2.044)	(0.002)	(0.003)	(0.003)	(0.001)	(0.003)	(0.001)
		VD	4.969	1.000	1.000	1.000	0.999	1.000	0.957
			(2.097)	(0.001)	(0.008)	(0.026)	(0.001)	(0.002)	(0.026)
(400,50,20)	(0.2,0.2)	RM	8.911	1.000	1.000	1.000	1.000	0.923	0.958
			(2.378)	(0.006)	(0.016)	(0.054)	(0.001)	(0.079)	(0.054)
		VD	8.410	1.000	1.000	1.000	0.984	1.000	0.870
			(2.380)	(0.001)	(0.008)	(0.003)	(0.033)	(0.001)	(0.077)
	(0.5,0.5)	RM	7.432	1.000	1.000	1.000	1.000	1.000	1.000
			(2.424)	(0.016)	(0.018)	(0.086)	(0.001)	(0.056)	(0.053)
		VD	8.016	1.000	1.000	1.000	1.000	0.923	0.958
			(2.420)	(0.015)	(0.013)	(0.016)	(0.003)	(0.099)	(0.095)
(300,500,400)	(0.2,0.2)	RM	7.674	1.000	1.000	1.000	1.000	0.985	0.993
			(2.101)	(0.001)	(0.004)	(0.001)	(0.001)	(0.025)	(0.027)
		VD	7.835	1.000	0.909	0.917	1.000	1.000	1.000
			(2.114)	(0.001)	(0.018)	(0.067)	(0.001)	(0.002)	(0.002)
	(0.5,0.5)	RM	7.883	1.000	0.977	0.992	1.000	0.985	0.993
			(2.338)	(0.001)	(0.032)	(0.017)	(0.001)	(0.009)	(0.008)
		VD	7.220	1.000	0.981	0.987	1.000	0.985	0.993
			(2.340)	(0.001)	(0.025)	(0.028)	(0.001)	(0.031)	(0.009)
(200,200,50)	(0.2,0.2)	RM	7.287	1.000	1.000	1.000	1.000	1.000	1.000
			(1.950)	(0.001)	(0.016)	(0.019)	(0.002)	(0.006)	(0.014)
		VD	7.062	1.000	1.000	1.000	1.000	1.000	1.000
			(2.035)	(0.001)	(0.004)	(0.004)	(0.002)	(0.012)	(0.004)
	(0.5,0.5)	RM	6.787	1.000	1.000	1.000	0.999	1.000	0.982
			(2.194)	(0.001)	(0.020)	(0.018)	(0.002)	(0.012)	(0.033)
		VD	6.413	1.000	1.000	1.000	0.999	1.000	0.982
			(2.203)	(0.001)	(0.006)	(0.005)	(0.001)	(0.003)	(0.062)

Table A.8: Simulation results under Scenario II, Setting A, and the independence structure in Section 3.3. Values in parentheses are Monte Carlo variances scaled by a factor of 100.

(n, p, m)	Error	Information	Variable Selection				Network Detection		
			$\|\Delta\boldsymbol{\Upsilon}\|_1$	SPE	SEN	MCC	SPE	SEN	MCC
(100,50,200)	(0.2,0.2)	RM	4.503	1.000	1.000	1.000	1.000	1.000	0.976
			(1.230)	(0.001)	(0.037)	(0.014)	(0.001)	(0.001)	(0.001)
		VD	4.104	1.000	1.000	1.000	1.000	1.000	1.000
			(1.255)	(0.001)	(0.002)	(0.002)	(0.001)	(0.001)	(0.001)
	(0.5,0.5)	RM	4.329	1.000	0.909	0.953	1.000	1.000	1.000
			(1.280)	(0.001)	(0.019)	(0.028)	(0.001)	(0.005)	(0.001)
		VD	3.821	1.000	1.000	1.000	1.000	1.000	1.000
			(1.343)	(0.001)	(0.003)	(0.003)	(0.001)	(0.001)	(0.001)
(400,50,20)	(0.2,0.2)	RM	6.301	1.000	1.000	1.000	1.000	1.000	1.000
			(1.234)	(0.009)	(0.041)	(0.052)	(0.001)	(0.001)	(0.043)
		VD	5.970	1.000	1.000	1.000	1.000	1.000	1.000
			(1.530)	(0.001)	(0.003)	(0.003)	(0.001)	(0.001)	(0.001)
	(0.5,0.5)	RM	6.334	1.000	1.000	1.000	1.000	1.000	1.000
			(1.246)	(0.007)	(0.047)	(0.038)	(0.001)	(0.017)	(0.018)
		VD	5.417	1.000	1.000	1.000	0.995	1.000	0.951
			(1.755)	(0.001)	(0.002)	(0.002)	(0.010)	(0.001)	(0.023)
(300,500,400)	(0.2,0.2)	RM	6.127	0.987	1.000	0.964	1.000	1.000	1.000
			(1.793)	(0.017)	(0.001)	(0.016)	(0.001)	(0.001)	(0.003)
		VD	6.136	1.000	1.000	1.000	1.000	1.000	1.000
			(1.911)	(0.001)	(0.008)	(0.002)	(0.001)	(0.001)	(0.003)
	(0.5,0.5)	RM	5.823	0.988	0.990	0.996	1.000	1.000	1.000
			(1.946)	(0.012)	(0.009)	(0.008)	(0.001)	(0.001)	(0.004)
		VD	4.935	1.000	1.000	1.000	1.000	1.000	1.000
			(2.011)	(0.001)	(0.003)	(0.003)	(0.001)	(0.001)	(0.001)
(200,200,50)	(0.2,0.2)	RM	5.758	1.000	1.000	1.000	0.999	1.000	0.980
			(1.369)	(0.001)	(0.013)	(0.013)	(0.015)	(0.002)	(0.033)
		VD	5.305	1.000	1.000	1.000	1.000	1.000	1.000
			(1.796)	(0.001)	(0.002)	(0.002)	(0.001)	(0.001)	(0.001)
	(0.5,0.5)	RM	5.052	1.000	0.909	0.953	1.000	1.000	1.000
			(1.770)	(0.001)	(0.078)	(0.054)	(0.002)	(0.019)	(0.013)
		VD	4.465	1.000	1.000	1.000	1.000	1.000	1.000
			(2.137)	(0.002)	(0.002)	(0.003)	(0.001)	(0.001)	(0.001)

Table A.9: Simulation results under Scenario I, Setting A, and the network structure in Section 3.3. Values in parentheses are Monte Carlo variances scaled by a factor of 100.

(n, p, m)	Error	Information	Variable Selection				Network Detection		
			$\|\Delta\boldsymbol{\Upsilon}\|_1$	SPE	SEN	MCC	SPE	SEN	MCC
(100,50,200)	(0.2,0.2)	RM	2.555	1.000	1.000	1.000	1.000	0.981	0.953
			(1.194)	(0.001)	(0.020)	(0.004)	(0.001)	(0.002)	(0.001)
		VD	2.416	1.000	1.000	1.000	1.000	0.981	0.953
			(1.288)	(0.001)	(0.002)	(0.002)	(0.001)	(0.003)	(0.020)
	(0.5,0.5)	RM	2.590	1.000	1.000	1.000	0.990	0.971	0.963
			(1.295)	(0.002)	(0.002)	(0.002)	(0.015)	(0.020)	(0.041)
		VD	2.326	1.000	1.000	0.957	1.000	1.000	1.000
			(1.303)	(0.001)	(0.004)	(0.006)	(0.001)	(0.001)	(0.001)
(400,50,20)	(0.2,0.2)	RM	3.047	1.000	1.000	1.000	0.979	0.946	0.972
			(1.121)	(0.010)	(0.001)	(0.059)	(0.001)	(0.029)	(0.053)
		VD	2.990	1.000	1.000	1.000	0.979	0.946	0.972
			(1.247)	(0.007)	(0.001)	(0.002)	(0.004)	(0.001)	(0.010)
	(0.5,0.5)	RM	3.022	0.999	1.000	0.957	0.952	1.000	0.960
			(1.203)	(0.012)	(0.001)	(0.028)	(0.099)	(0.005)	(0.021)
		VD	2.989	0.999	1.000	0.957	0.968	1.000	0.955
			(1.449)	(0.023)	(0.001)	(0.050)	(0.005)	(0.001)	(0.018)
(300,500,400)	(0.2,0.2)	RM	2.923	0.987	1.000	0.965	1.000	0.985	0.993
			(2.086)	(0.006)	(0.001)	(0.005)	(0.001)	(0.020)	(0.015)
		VD	2.889	0.987	1.000	0.965	1.000	0.985	0.993
			(2.094)	(0.006)	(0.001)	(0.006)	(0.001)	(0.025)	(0.011)
	(0.5,0.5)	RM	3.095	0.987	1.000	0.964	1.000	0.985	0.993
			(2.101)	(0.005)	(0.001)	(0.008)	(0.001)	(0.026)	(0.009)
		VD	2.834	0.988	1.000	0.966	1.000	0.985	0.993
			(2.105)	(0.004)	(0.001)	(0.007)	(0.001)	(0.035)	(0.007)
(200,200,50)	(0.2,0.2)	RM	2.837	1.000	1.000	1.000	0.997	0.992	0.985
			(1.533)	(0.001)	(0.001)	(0.001)	(0.002)	(0.006)	(0.003)
		VD	2.623	1.000	1.000	1.000	0.997	0.992	0.958
			(1.679)	(0.003)	(0.003)	(0.003)	(0.001)	(0.008)	(0.010)
	(0.5,0.5)	RM	2.904	1.000	1.000	1.000	0.961	0.993	0.953
			(1.702)	(0.001)	(0.002)	(0.010)	(0.001)	(0.006)	(0.006)
		VD	2.731	1.000	1.000	1.000	0.962	1.000	0.950
			(1.729)	(0.001)	(0.002)	(0.003)	(0.012)	(0.003)	(0.0014)

Table A.10: Simulation results under Scenario I, Setting A, and the independence structure in Section 3.3. Values in parentheses are Monte Carlo variances scaled by a factor of 100.

(n,p,m)	Error	Information	Variable Selection				Network Detection		
			$\|\Delta\boldsymbol{\Upsilon}\|_1$	SPE	SEN	MCC	SPE	SEN	MCC
(100,50,200)	(0.2,0.2)	RM	2.633	1.000	1.000	1.000	1.000	1.000	0.958
			(1.413)	(0.001)	(0.014)	(0.001)	(0.001)	(0.001)	(0.002)
		VD	2.666	1.000	1.000	1.000	1.000	0.990	0.962
			(1.505)	(0.001)	(0.005)	(0.015)	(0.001)	(0.001)	(0.002)
	(0.5,0.5)	RM	2.593	1.000	1.000	1.000	1.000	1.000	0.990
			(1.489)	(0.002)	(0.003)	(0.003)	(0.001)	(0.001)	(0.002)
		VD	2.506	1.000	1.000	1.000	0.990	0.990	0.956
			(1.622)	(0.001)	(0.002)	(0.004)	(0.001)	(0.001)	(0.002)
(400,50,20)	(0.2,0.2)	RM	3.202	1.000	1.000	1.000	0.989	1.000	0.908
			(1.280)	(0.009)	(0.001)	(0.004)	(0.005)	(0.001)	(0.019)
		VD	3.170	1.000	1.000	1.000	0.989	1.000	0.908
			(1.345)	(0.010)	(0.001)	(0.040)	(0.008)	(0.001)	(0.022)
	(0.5,0.5)	RM	3.322	0.999	1.000	0.957	0.995	1.000	0.951
			(1.517)	(0.002)	(0.001)	(0.034)	(0.005)	(0.001)	(0.021)
		VD	3.078	1.000	1.000	1.000	1.000	1.000	1.000
			(1.690)	(0.002)	(0.003)	(0.027)	(0.005)	(0.001)	(0.019)
(300,500,400)	(0.2,0.2)	RM	3.083	0.987	1.000	0.965	1.000	1.000	1.000
			(1.663)	(0.015)	(0.001)	(0.011)	(0.001)	(0.001)	(0.002)
		VD	3.050	0.987	1.000	0.964	1.000	0.985	0.993
			(1.678)	(0.023)	(0.001)	(0.013)	(0.001)	(0.001)	(0.003)
	(0.5,0.5)	RM	3.070	0.987	1.000	0.966	1.000	1.000	1.000
			(1.850)	(0.013)	(0.001)	(0.015)	(0.001)	(0.001)	(0.001)
		VD	2.924	0.987	1.000	0.964	1.000	1.000	1.000
			(1.947)	(0.015)	(0.001)	(0.013)	(0.001)	(0.001)	(0.002)
(200,200,50)	(0.2,0.2)	RM	2.927	1.000	1.000	1.000	0.998	1.000	0.961
			(1.660)	(0.002)	(0.001)	(0.001)	(0.003)	(0.001)	(0.013)
		VD	2.908	1.000	1.000	1.000	0.998	0.992	0.972
			(1.719)	(0.001)	(0.002)	(0.007)	(0.001)	(0.001)	(0.055)
	(0.5,0.5)	RM	2.887	1.000	1.000	1.000	0.998	1.000	0.961
			(1.711)	(0.001)	(0.006)	(0.002)	(0.002)	(0.001)	(0.049)
		VD	2.774	1.000	1.000	1.000	0.998	1.000	0.961
			(1.894)	(0.001)	(0.004)	(0.009)	(0.002)	(0.001)	(0.073)

Table A.11: Simulation results under Scenario II, Setting B, and the network structure in Section 3.3. Values in parentheses are Monte Carlo variances scaled by a factor of 100.

(n, p, m)	Error	Information	Variable Selection				Network Detection		
			$\|\Delta\boldsymbol{\Upsilon}\|_1$	SPE	SEN	MCC	SPE	SEN	MCC
(100,50,200)	(0.2,0.2)	RM	4.783	1.000	1.000	1.000	1.000	1.000	1.000
			(1.933)	(0.018)	(1.122)	(0.237)	(0.143)	(0.056)	(0.081)
		VD	4.694	1.000	1.000	1.000	0.995	1.000	0.961
			(1.810)	(0.002)	(0.002)	(0.001)	(0.001)	(0.029)	(0.025)
	(0.5,0.5)	RM	4.822	1.000	1.000	1.000	1.000	1.000	1.000
			(2.023)	(0.046)	(0.619)	(0.100)	(0.069)	(0.154)	(0.060)
		VD	4.850	1.000	1.000	1.000	0.996	1.000	0.973
			(2.100)	(0.003)	(0.002)	(0.002)	(0.009)	(0.001)	(0.018)
(400,50,20)	(0.2,0.2)	RM	6.433	1.000	1.000	1.000	1.000	0.923	0.958
			(2.400)	(0.005)	(0.078)	(0.027)	(0.206)	(0.056)	(1.243)
		VD	8.471	1.000	1.000	1.000	1.000	1.000	1.000
			(2.360)	(0.006)	(0.011)	(0.005)	(0.039)	(0.049)	(0.043)
	(0.5,0.5)	RM	6.568	0.960	1.000	0.938	1.000	0.923	0.958
			(2.433)	(0.048)	(0.139)	(0.090)	(0.587)	(0.000)	(0.131)
		VD	8.313	1.000	1.000	1.000	0.960	1.000	0.958
			(2.410)	(0.006)	(0.013)	(0.016)	(0.044)	(0.029)	(0.086)
(300,500,400)	(0.2,0.2)	RM	7.558	1.000	0.990	0.988	1.000	0.995	0.998
			(2.030)	(0.001)	(0.407)	(0.001)	(0.010)	(0.001)	(0.016)
		VD	7.495	1.000	0.990	0.953	0.997	1.000	0.958
			(2.123)	(0.003)	(0.072)	(0.008)	(0.010)	(0.004)	(0.040)
	(0.5,0.5)	RM	7.780	1.000	0.988	0.987	1.000	0.995	0.990
			(2.340)	(0.001)	(0.073)	(0.001)	(0.001)	(0.001)	(0.001)
		VD	7.656	1.000	0.981	0.987	1.000	0.975	0.960
			(2.345)	(0.002)	(0.029)	(0.058)	(0.001)	(0.025)	(0.025)
(200,200,50)	(0.2,0.2)	RM	6.893	1.000	1.000	1.000	0.971	1.000	0.965
			(1.943)	(0.001)	(0.775)	(0.019)	(0.099)	(0.031)	(0.092)
		VD	6.988	1.000	1.000	1.000	0.983	0.964	0.988
			(2.303)	(0.016)	(0.014)	(0.007)	(0.050)	(0.029)	(0.008)
	(0.5,0.5)	RM	6.843	1.000	1.000	1.000	1.000	0.995	0.993
			(2.223)	(0.002)	(0.004)	(0.001)	(0.001)	(0.002)	(0.001)
		VD	6.644	1.000	1.000	1.000	0.960	1.000	0.983
			(2.388)	(0.006)	(0.014)	(0.016)	(0.441)	(0.030)	(0.086)

Table A.12: Simulation results under Scenario II, Setting B, and the independence structure in Section 3.3. Values in parentheses are Monte Carlo variances scaled by a factor of 100.

(n, p, m)	Error	Information	Variable Selection				Network Detection		
			$\|\Delta\boldsymbol{\Upsilon}\|_1$	SPE	SEN	MCC	SPE	SEN	MCC
(100,50,200)	(0.2,0.2)	RM	3.890	1.000	1.000	1.000	1.000	1.000	0.956
			(1.137)	(0.006)	(0.460)	(0.054)	(0.181)	(0.491)	(0.205)
		VD	4.113	1.000	1.000	1.000	0.995	1.000	0.951
			(1.250)	(0.022)	(0.008)	(0.014)	(0.029)	(0.001)	(0.059)
	(0.5,0.5)	RM	4.115	1.000	1.000	1.000	1.000	1.000	1.000
			(1.258)	(0.023)	(1.037)	(0.148)	(0.190)	(0.399)	(0.188)
		VD	4.093	1.000	0.990	0.975	0.991	1.000	0.981
			(1.355)	(0.003)	(0.049)	(0.018)	(0.010)	(0.001)	(0.014)
(400,50,20)	(0.2,0.2)	RM	6.123	1.000	1.000	1.000	0.995	1.000	0.986
			(1.200)	(0.006)	(0.041)	(0.050)	(0.047)	(0.001)	(0.350)
		VD	5.844	1.000	1.000	1.000	1.000	1.000	1.000
			(1.566)	(0.007)	(0.008)	(0.005)	(0.017)	(0.000)	(0.023)
	(0.5,0.5)	RM	6.158	1.000	1.000	1.000	0.995	1.000	0.960
			(1.231)	(0.019)	(0.001)	(0.046)	(0.468)	(0.267)	(0.190)
		VD	6.220	1.000	1.000	1.000	0.979	1.000	0.986
			(1.830)	(0.002)	(0.004)	(0.004)	(0.064)	(0.001)	(0.012)
(300,500,400)	(0.2,0.2)	RM	6.078	1.000	1.000	1.000	1.000	0.985	0.990
			(1.890)	(0.001)	(0.473)	(0.001)	(0.005)	(0.001)	(0.006)
		VD	6.105	1.000	0.990	0.973	0.996	1.000	0.990
			(1.903)	(0.001)	(0.034)	(0.007)	(0.001)	(0.027)	(0.030)
	(0.5,0.5)	RM	5.905	1.000	1.000	1.000	1.000	0.990	0.993
			(2.030)	(0.003)	(0.473)	(0.001)	(0.001)	(0.001)	(0.001)
		VD	6.202	1.000	1.000	1.000	1.000	0.952	0.974
			(2.145)	(0.002)	(0.004)	(0.001)	(0.001)	(0.009)	(0.005)
(200,200,50)	(0.2,0.2)	RM	5.063	1.000	1.000	1.000	0.979	1.000	0.965
			(1.355)	(0.001)	(0.357)	(0.011)	(0.083)	(0.031)	(0.057)
		VD	5.633	0.999	1.000	0.980	0.992	1.000	0.982
			(2.356)	(0.015)	(0.022)	(0.032)	(0.067)	(0.001)	(0.065)
	(0.5,0.5)	RM	5.104	0.960	1.000	0.958	1.000	1.000	1.000
			(1.743)	(0.058)	(0.001)	(0.043)	(0.003)	(0.001)	(0.002)
		VD	5.288	1.000	1.000	0.998	0.998	1.000	0.994
			(2.420)	(0.017)	(0.010)	(0.062)	(0.079)	(0.001)	(0.007)

Table A.13: Simulation results under Scenario I, Setting B, and the network structure in Section 3.3. Values in parentheses are Monte Carlo variances scaled by a factor of 100.

(n,p,m)	Error	Information	Variable Selection				Network Detection		
			$\|\Delta\boldsymbol{\Upsilon}\|_1$	SPE	SEN	MCC	SPE	SEN	MCC
(100,50,200)	(0.2,0.2)	RM	2.488	1.000	1.000	1.000	1.000	0.981	0.962
			(1.200)	(0.003)	(0.041)	(0.003)	(0.002)	(0.005)	(0.001)
		VD	2.347	1.000	1.000	1.000	1.000	0.971	0.980
			(1.358)	(0.012)	(0.013)	(0.006)	(0.001)	(0.004)	(0.001)
	(0.5,0.5)	RM	2.520	1.000	1.000	1.000	0.964	0.971	0.952
			(1.310)	(0.068)	(0.139)	(0.011)	(0.001)	(0.002)	(0.001)
		VD	2.437	1.000	1.000	1.000	1.000	0.971	0.980
			(1.449)	(0.014)	(0.020)	(0.012)	(0.001)	(0.003)	(0.001)
(400,50,20)	(0.2,0.2)	RM	3.054	1.000	1.000	1.000	0.965	0.946	0.973
			(1.180)	(0.008)	(0.001)	(0.036)	(0.551)	(0.080)	(0.036)
		VD	3.114	1.000	1.000	1.000	0.984	0.967	0.975
			(1.255)	(0.019)	(0.001)	(0.018)	(0.040)	(0.013)	(0.030)
	(0.5,0.5)	RM	3.020	1.000	1.000	1.000	0.981	0.967	0.972
			(1.193)	(0.066)	(0.001)	(0.089)	(0.178)	(0.056)	(0.093)
		VD	3.159	0.989	1.000	0.974	0.973	0.976	0.965
			(1.567)	(0.081)	(0.001)	(0.012)	(0.020)	(0.012)	(0.013)
(300,500,400)	(0.2,0.2)	RM	2.960	0.993	1.000	0.985	1.000	0.985	0.990
			(2.103)	(0.023)	(0.014)	(0.033)	(0.002)	(0.035)	(0.027)
		VD	2.955	1.000	1.000	1.000	1.000	0.973	0.971
			(2.105)	(0.002)	(0.001)	(0.003)	(0.02)	(0.039)	(0.037)
	(0.5,0.5)	RM	3.114	1.000	0.964	0.953	1.000	0.985	0.993
			(2.022)	(0.002)	(0.034)	(0.040)	(0.001)	(0.022)	(0.015)
		VD	3.122	1.000	1.000	1.000	1.000	0.987	0.983
			(2.120)	(0.002)	(0.001)	(0.003)	(0.002)	(0.024)	(0.018)
(200,200,50)	(0.2,0.2)	RM	2.808	1.000	1.000	1.000	0.988	0.992	0.983
			(1.500)	(0.013)	(0.001)	(0.014)	(0.200)	(0.029)	(0.040)
		VD	2.645	0.960	1.000	0.938	0.996	0.983	0.958
			(1.740)	(0.027)	(0.001)	(0.025)	(0.015)	(0.035)	(0.024)
	(0.5,0.5)	RM	2.856	1.000	1.000	1.000	0.968	1.000	0.960
			(1.640)	(0.004)	(0.001)	(0.001)	(0.032)	(0.001)	(0.030)
		VD	2.871	0.990	1.000	0.972	0.993	0.983	0.988
			(1.800)	(0.062)	(0.017)	(0.011)	(0.030)	(0.043)	(0.006)

Table A.14: Simulation results under Scenario I, Setting B, and the independence structure in Section 3.3. Values in parentheses are Monte Carlo variances scaled by a factor of 100.

(n, p, m)	Error	Information	Variable Selection				Network Detection		
			$\|\Delta\boldsymbol{\Upsilon}\|_1$	SPE	SEN	MCC	SPE	SEN	MCC
(100,50,200)	(0.2,0.2)	RM	2.479	1.000	1.000	1.000	1.000	1.000	1.000
			(1.340)	(0.002)	(0.078)	(0.002)	(0.004)	(0.001)	(0.001)
		VD	2.598	0.984	1.000	0.987	0.993	1.000	0.985
			(1.530)	(0.056)	(0.002)	(0.017)	(0.024)	(0.001)	(0.002)
	(0.5,0.5)	RM	2.343	1.000	1.000	1.000	1.000	1.000	1.000
			(1.500)	(0.059)	(0.011)	(0.009)	(0.007)	(0.001)	(0.002)
		VD	2.603	0.964	1.000	0.953	1.000	1.000	0.967
			(1.678)	(0.047)	(0.003)	(0.014)	(0.001)	(0.001)	(0.001)
(400,50,20)	(0.2,0.2)	RM	2.233	1.000	1.000	1.000	1.000	1.000	1.000
			(1.230)	(0.014)	(0.001)	(0.068)	(0.078)	(0.001)	(0.028)
		VD	2.538	1.000	1.000	1.000	0.979	1.000	0.984
			(1.355)	(0.019)	(0.001)	(0.005)	(0.039)	(0.001)	(0.013)
	(0.5,0.5)	RM	2.367	1.000	1.000	1.000	1.000	1.000	1.000
			(1.500)	(0.067)	(0.001)	(0.093)	(0.016)	(0.001)	(0.019)
		VD	2.766	1.000	1.000	1.000	1.000	1.000	1.000
			(1.750)	(0.007)	(0.001)	(0.001)	(0.002)	(0.002)	(0.001)
(300,500,400)	(0.2,0.2)	RM	2.533	1.000	0.972	0.986	1.000	1.000	1.000
			(1.780)	(0.002)	(0.036)	(0.029)	(0.002)	(0.001)	(0.001)
		VD	2.450	1.000	1.000	1.000	1.000	0.975	0.981
			(1.766)	(0.003)	(0.001)	(0.004)	(0.002)	(0.026)	(0.018)
	(0.5,0.5)	RM	2.388	1.000	1.000	1.000	1.000	1.000	0.995
			(1.933)	(0.002)	(0.001)	(0.002)	(0.001)	(0.001)	(0.001)
		VD	2.466	1.000	1.000	1.000	0.991	1.000	0.987
			(2.023)	(0.003)	(0.001)	(0.005)	(0.034)	(0.001)	(0.010)
(200,200,50)	(0.2,0.2)	RM	2.660	1.000	1.000	1.000	1.000	1.000	1.000
			(1.640)	(0.026)	(0.001)	(0.028)	(0.013)	(0.001)	(0.016)
		VD	2.780	0.988	1.000	0.992	0.993	1.000	0.986
			(1.740)	(0.027)	(0.001)	(0.025)	(0.059)	(0.001)	(0.012)
	(0.5,0.5)	RM	3.104	1.000	0.990	0.989	1.000	1.000	1.000
			(1.700)	(0.031)	(0.058)	(0.015)	(0.011)	(0.006)	(0.006)
		VD	2.942	1.000	1.000	1.000	0.993	1.000	0.985
			(2.030)	(0.006)	(0.001)	(0.005)	(0.055)	(0.017)	(0.017)

Appendix B Additional Results for Real Data Analysis

In this appendix, we summarize the result of GBM data analysis. Specifically,

- Figures B.1-B.5 display the relationships between gene expressions and microRNAs under Scenarios I and II.
- Figures B.6-B.9 display the relationships of pairs of microRNAs under Scenarios I and II.

The interpretation of the above figures can be found in the main text.

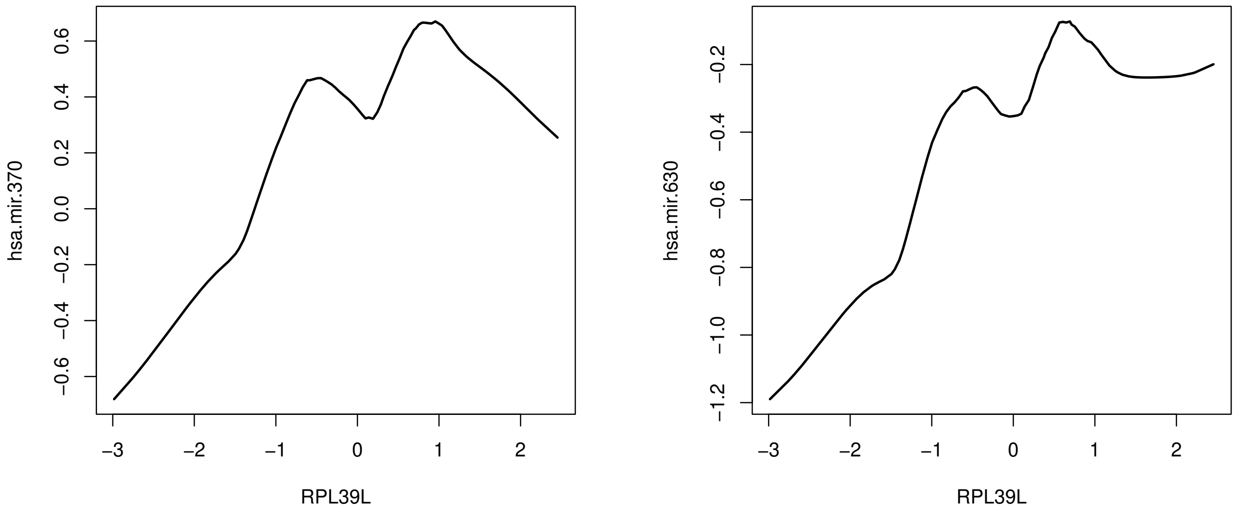

Figure B.1: Real data analysis results: Model fitting of a gene RPL39L and two microRNAs (hsa.mir.370 and hsa.mir.630) under $\sigma_\delta = \sigma_\eta = 0.2$ in Scenario I.

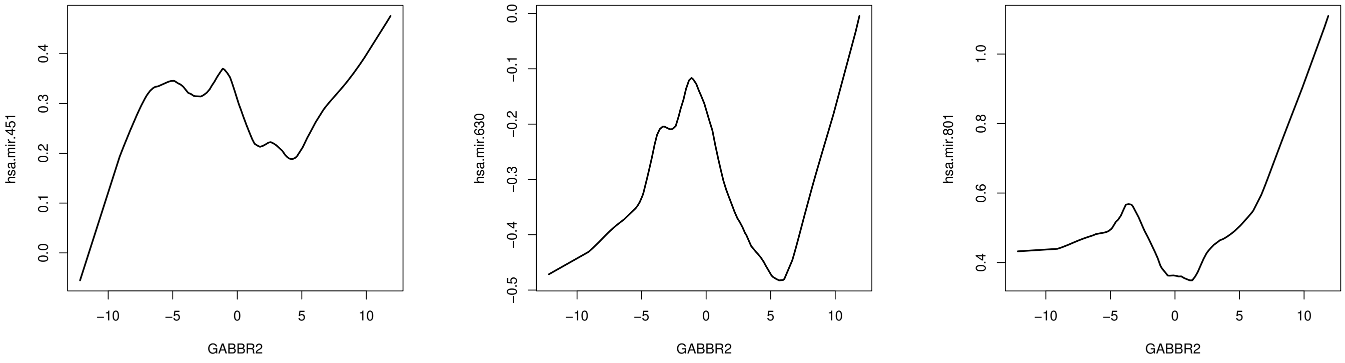

Figure B.2: Real data analysis results: Model fitting of a gene GABBR2 and three microRNAs (hsa.mir.451, hsa.mir.630, and hsa.mir.801) $\sigma_\delta = \sigma_\eta = 0.5$ in Scenario I.

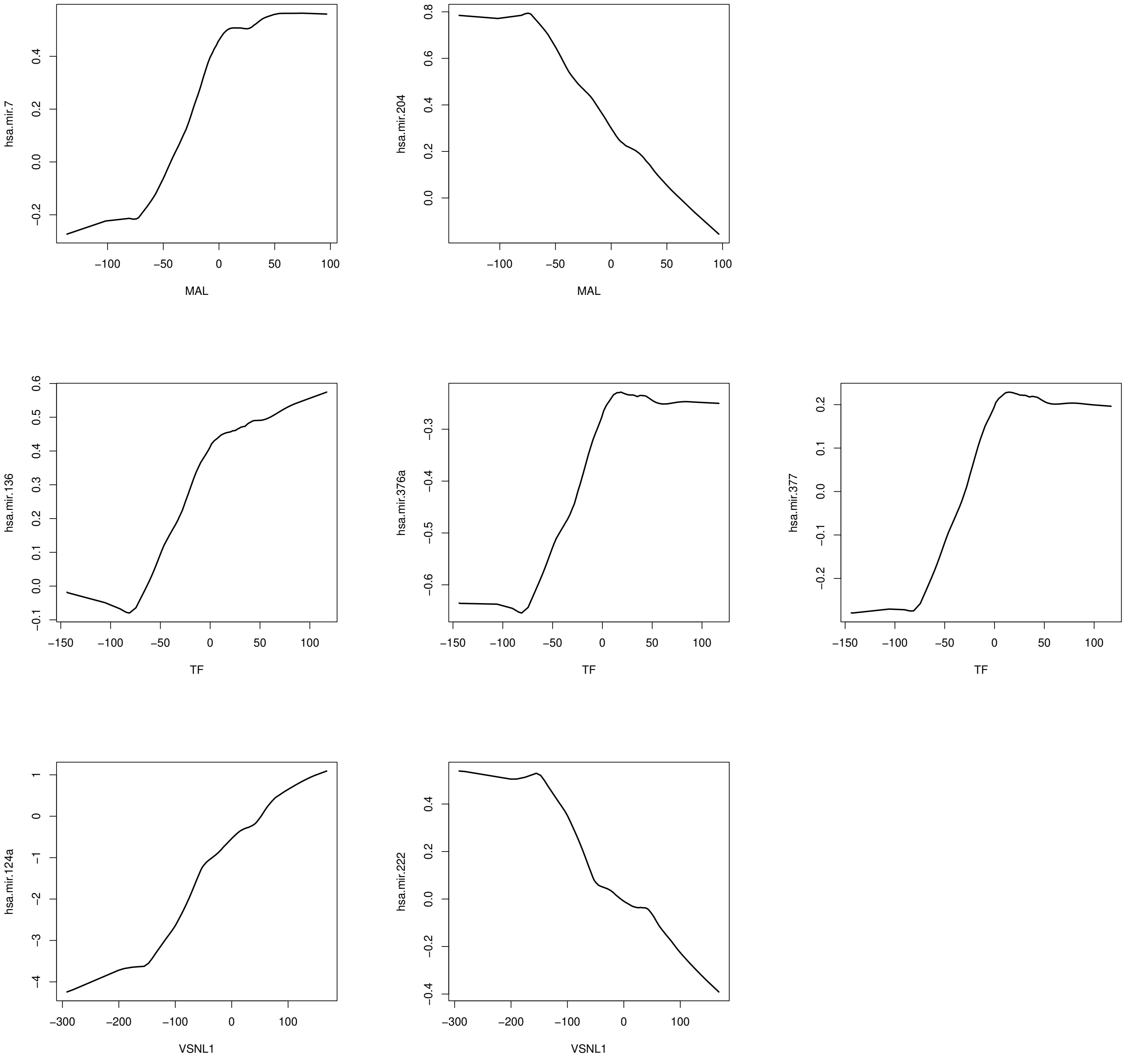

Figure B.3: Real data analysis results: Model fitting of three genes (MAL, TF, and VSNL1) and several microRNAs (hsa.mir.7, hsa.mir.204, hsa.mir.136, hsa.mir.376a, hsa.mir.377, hsa.mir.124a, and hsa.mir.222) under $R_W = R_Z = 0.5$ in Scenario II.

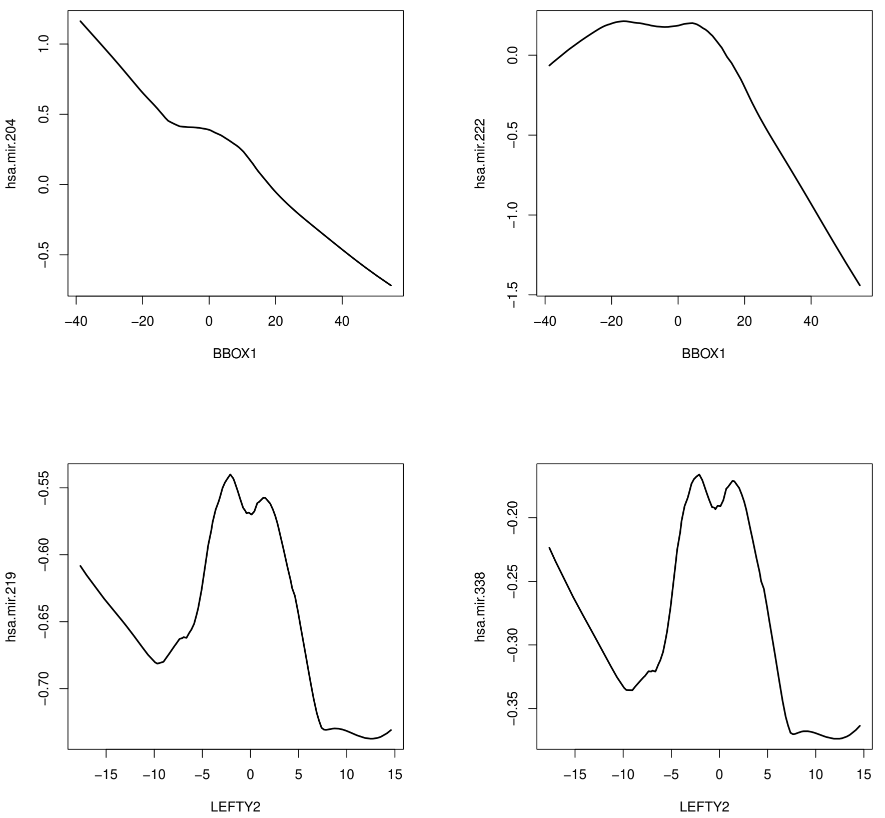

Figure B.4: Real data analysis results: Model fitting of two genes (BBOX1 and LEFTY2) and four microRNAs (hsa.mir.204, hsa.mir.222, hsa.mir.219, and hsa.mir.338) under $R_W = R_Z = 0.8$ in Scenario II.

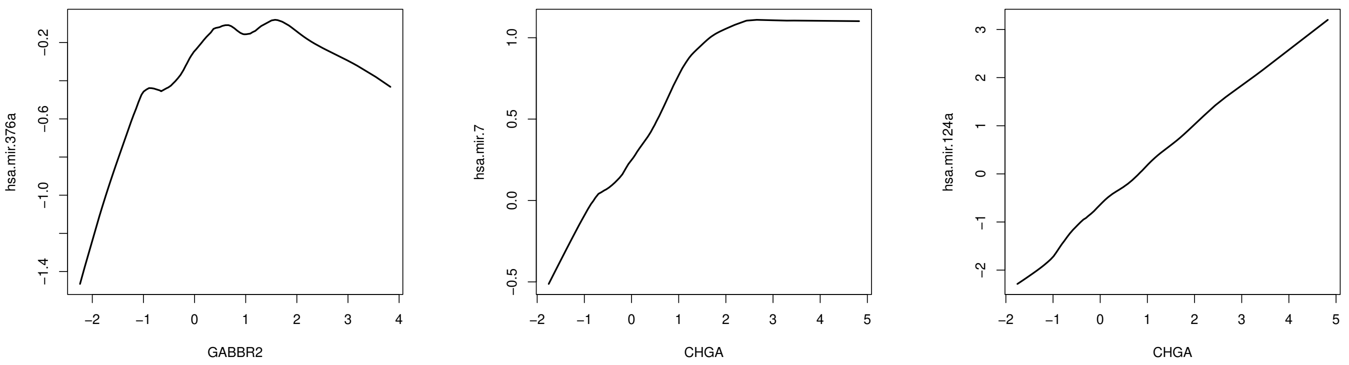

Figure B.5: Real data analysis results: Model fitting of two genes (GABBR2 and CHGA) and three microRNAs (hsa.mir.376a, hsa.mir.7, and hsa.mir.124a) determined by the naive method.

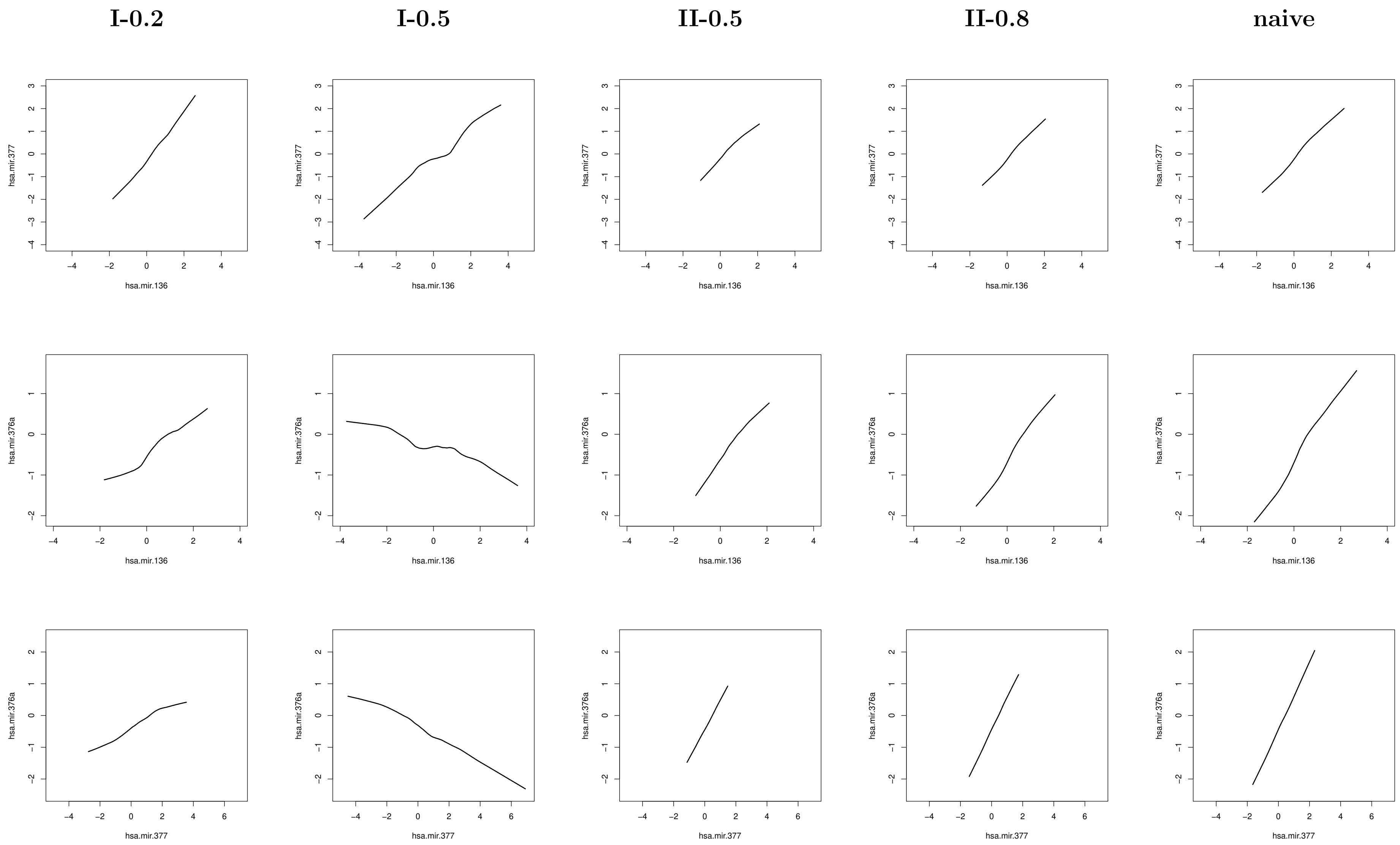

Figure B.6: Real data analysis results: Model fitting of the pairs of microRNAs commonly selected across different scenarios. The caption I-y indicates Scenario I with $\sigma_\eta = \sigma_\delta = y$ for $y = 0.2$ or 0.5; the caption II-r indicates Scenario II with $R_W = R_Z = r$ for $r = 0.5$ or 0.8; the caption naive reflects $\sigma_\eta = \sigma_\delta = 0$.

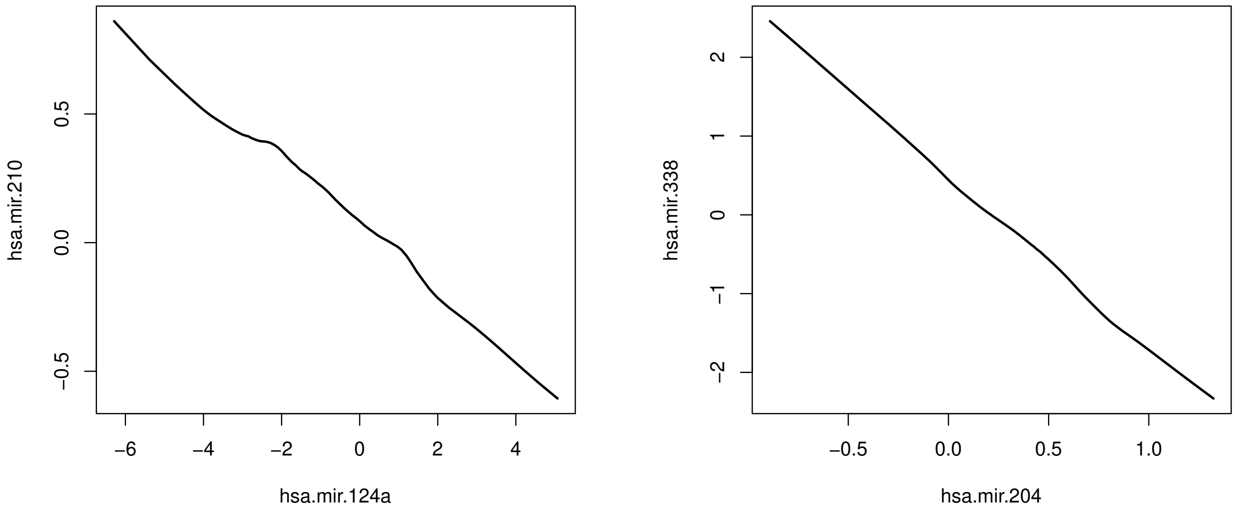

Figure B.7: Real data analysis results: Model fitting of two pairs (hsa.mir.124a, hsa.mir.210) and (hsa.mir.204, hsa.mir.338) uniquely selected under under $R_W = R_Z = 0.8$ in Scenario II.

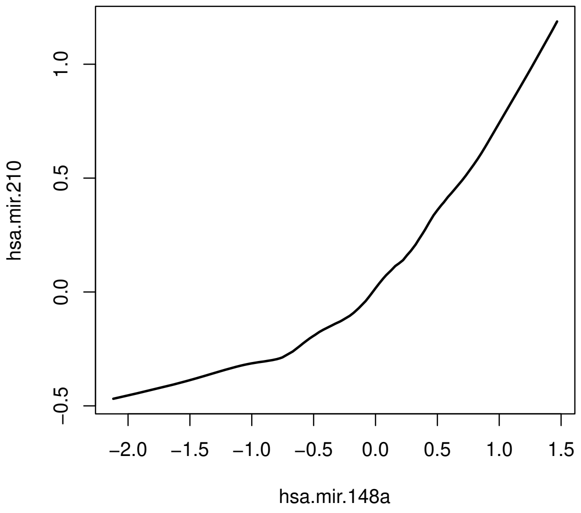

Figure B.8: Real data analysis results: Model fitting of the pair (hsa.mir.630, hsa.mir.7) uniquely selected under $\sigma_\delta = \sigma_\eta = 0.5$ in Scenario I.

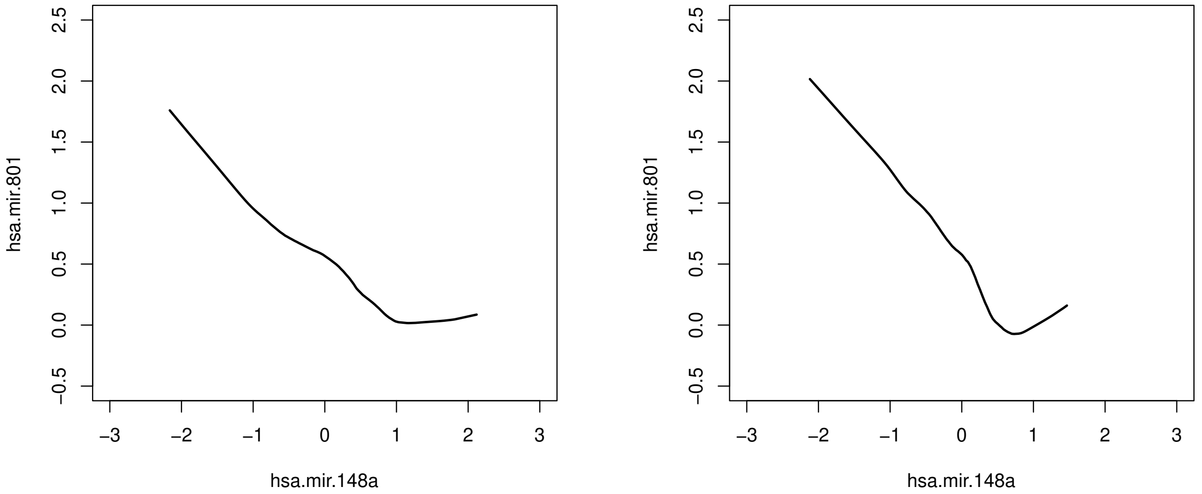

Figure B.9: Real data analysis results: Model fitting of a pair (hsa.mir.148a, hsa.mir.801) selected under under $\sigma_\delta = \sigma_\eta = 0.2$ (left penal) and 0.5 (right panel) in Scenario I.
